# Causal role of high body mass index in multiple chronic diseases: a systematic review and meta-analysis of Mendelian randomization studies

**DOI:** 10.1186/s12916-021-02188-x

**Published:** 2021-12-15

**Authors:** Susanna C. Larsson, Stephen Burgess

**Affiliations:** 1 Unit of Medical Epidemiology, Department of Surgical Sciences, Uppsala University, Uppsala, Sweden; 2 Unit of Cardiovascular and Nutritional Epidemiology, Institute of Environmental Medicine, Karolinska Institutet, Stockholm, Sweden; 3 Department of Public Health and Primary Care, University of Cambridge, Cambridge, UK; 4 MRC Biostatistics Unit, University of Cambridge, Cambridge, UK

**Keywords:** Body mass index, Cancer, Cardiovascular disease, Chronic diseases, Obesity

## Abstract

**Background:**

Obesity is a worldwide epidemic that has been associated with a plurality of diseases in observational studies. The aim of this study was to summarize the evidence from Mendelian randomization (MR) studies of the association between body mass index (BMI) and chronic diseases.

**Methods:**

PubMed and Embase were searched for MR studies on adult BMI in relation to major chronic diseases, including diabetes mellitus; diseases of the circulatory, respiratory, digestive, musculoskeletal, and nervous systems; and neoplasms. A meta-analysis was performed for each disease by using results from published MR studies and corresponding de novo analyses based on summary-level genetic data from the FinnGen consortium (*n* = 218,792 individuals).

**Results:**

In a meta-analysis of results from published MR studies and de novo analyses of the FinnGen consortium, genetically predicted higher BMI was associated with increased risk of type 2 diabetes mellitus, 14 circulatory disease outcomes, asthma, chronic obstructive pulmonary disease, five digestive system diseases, three musculoskeletal system diseases, and multiple sclerosis as well as cancers of the digestive system (six cancer sites), uterus, kidney, and bladder. In contrast, genetically predicted higher adult BMI was associated with a decreased risk of Dupuytren’s disease, osteoporosis, and breast, prostate, and non-melanoma cancer, and not associated with Alzheimer’s disease, amyotrophic lateral sclerosis, or Parkinson’s disease.

**Conclusions:**

The totality of the evidence from MR studies supports a causal role of excess adiposity in a plurality of chronic diseases. Hence, continued efforts to reduce the prevalence of overweight and obesity are a major public health goal.

**Supplementary Information:**

The online version contains supplementary material available at 10.1186/s12916-021-02188-x.

## Background

Obesity is a worldwide epidemic that has been associated with an increased risk of a plurality of chronic diseases in traditional observational studies [1–6]. These observational findings [1–6] may represent the causal effect of obesity on disease risk or confounding from other risk factors, such as a poor diet and physical inactivity. During the last years, an increasing number of Mendelian randomization (MR) studies of adiposity, mostly defined by body mass index (BMI), in relation to chronic diseases have been published [7–53]. MR is an instrumental variable analysis that exploits genetic variants with a robust impact on the exposure (e.g., BMI) as proxy markers for the exposure to test whether the exposure has a causal relationship with disease risk [54]. Compared with conventional observational studies, MR studies are less susceptible to confounding as genes are randomly assorted when passed from parents to descendants [54]. Additionally, MR studies are not biased by reverse causality as genes are constant and not modified by disease development.

The aim of this study was to conduct a systematic review and meta-analyses of MR studies to determine the causal role of excess adiposity in chronic diseases. Meta-analyses were performed using results from published MR studies and complemented with results from de novo MR analyses of pertinent disease outcomes in the FinnGen consortium.

## Methods

### Literature search and inclusion criteria

A search in the PubMed and Embase databases using the query “(Mendelian randomization) AND (body mass index OR overweight OR obesity or adiposity)” was performed on October 3, 2021. Eligible for inclusion were original articles that reported estimates from an MR analysis of genetically predicted adulthood BMI in relation to one or more chronic diseases in the following disease groups: diabetes mellitus (type 1 or type 2); disease of the circulatory, respiratory, digestive, musculoskeletal, or nervous system; or site-specific cancer. When more than one study was published on the same outcome and study population, the study based on the largest number of cases or the largest number of genetic variants (if the sample size was the same) was included. No restriction based on the number of cases was imposed.

### Data extraction and quality assessment

Data were extracted and entered in predefined tables by one author (SCL) and independently reviewed by another author (SB). From each MR study, the following information was extracted: the last name of the first author and year of publication; the number of single-nucleotide polymorphisms (SNPs) used as instrumental variables in the analysis, the source for the SNPs, and the exposure unit; consortium, study, or studies from which the SNP-outcome association estimates were obtained; the number of cases and non-cases or the total number of participants; ancestry of the study population; and the relative risk estimate (generally odds ratio [*OR*]) with 95% confidence interval (*CI*) for the BMI-outcome association from the primary analysis. The study quality was assessed by adapting a modified version of the Strengthening the Reporting of Mendelian Randomization Studies (STROBE-MR) Guidelines [55, 56].

### Statistical analysis

In most studies, the relative risk estimate was expressed per 1 standard deviation (*SD*; ~4.8 kg/m^2^) increase in genetically predicted BMI. For studies using another unit (e.g., 1 kg/m^2^), the estimate was rescaled to 1 *SD* increase in BMI. Meta-analysis was performed for each outcome using results of published MR studies and de novo MR analyses of summary-level genetic data from the R5 release of the FinnGen consortium (*n* = 218,792 individuals) [57]. For the de novo MR analyses of FinnGen data, independent SNPs (low linkage disequilibrium *R*^2^ < 0.001) associated with BMI at *P* < 5 × 10^−8^ in a genome-wide association meta-analysis of the Genetic Investigation of ANthropometric Traits consortium and the UK Biobank (*n* = 806,810 individuals) [58] were obtained from a recent MR study [49]. All BMI-associated SNPs were harmonized with the outcome data in FinnGen to ensure that effect estimates of each SNP on BMI and the outcome corresponded to the same effect allele. Analyses of FinnGen data were performed for all relevant diseases except aortic valve stenosis and osteoarthritis which were not available in the FinnGen database. Considering potential differential associations of BMI with disease risk in populations of different origins, sensitivity analyses confined to data based on European populations were conducted. Meta-analyses of results in non-European populations were not possible due to a lack of data from more than one study on the same disease. Heterogeneity between studies was quantified using the *I*^2^ statistic [59]. Values <25%, 25–75%, and >75% were considered low, moderate, and high heterogeneity. All statistical analyses were conducted in Stata (StataCorp, College Station, TX, USA) using the mrrobust and metan commands.

## Results

### Literature search and study selection

**Fig. 1:**
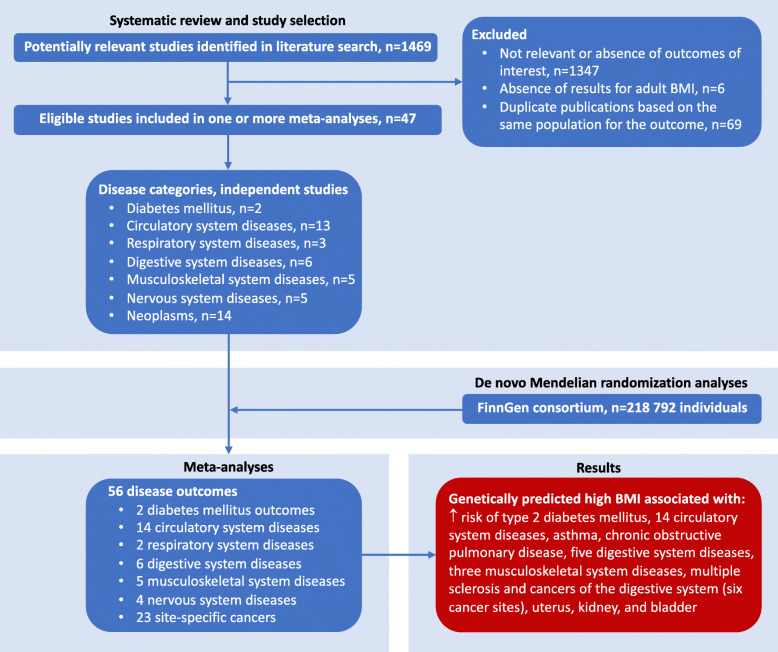
Flowchart of the study selection

The PubMed and Embase searches resulted in 1469 unique hits of which 116 articles reported results from an MR study of BMI in relation to one or more of the pertinent disease outcomes. An overview of study selection is presented in Fig. 1.

### Study quality and description

The results from the evaluation of study quality are provided in Additional file 1: Table S1. All studies indicated Mendelian randomization in the title and/or abstract, provided a rationale for the study and the objective, and provided information on the data sources used. In two studies, information on the number of cases and non-cases for the disease outcome(s) was not found [25, 29, 30]. Most studies obtained genetic instruments for BMI from a genome-wide association study based on the Genetic Investigation of ANthropometric Traits consortium with or without UK Biobank [58, 60, 61] and used 14 to several hundred SNPs as instrumental variables. The remaining studies used one to few selected SNPs in relevant obesity loci (e.g., *FTO*). Most recent MR studies used a strict linkage disequilibrium cut-off (*R*^2^ < 0.001) to select independent SNPs as instrumental variables for BMI, but some studies selected all conditional independent SNPs identified in the genome-wide association study. The majority of MR studies were based on outcome data from one or few studies (e.g., Danish, Swedish, and Chinese cohorts), a large-scale genetic consortium, or the UK Biobank. For several outcomes, two or more studies were published based on outcome data from the same source population (e.g., same consortium or UK Biobank). One two-sample MR study did not indicate the statistical method used for the primary analysis [14], and eight studies did report results of sensitivity analyses based on robust MR methods (e.g., weighted median and MR-Egger regression) [9, 11, 17, 24, 25, 35, 40, 41].

The number of MR studies based on independent study samples for each disease category was two for diabetes mellitus [7, 8], 13 for circulatory system diseases [9–21], three for respiratory diseases [8, 22, 23], six for digestive system diseases [24–29], five for musculoskeletal system diseases [30–34] plus FinnGen consortium (for osteoporosis), five for nervous system diseases [35–39], and 14 for neoplasms [40–53]. Among the selected studies, six studies included East-Asian (Chinese [11, 25, 42] and Japanese [48, 50]) or Chilean [53] individuals, whereas the remaining studies included individuals of European ancestry or mixed (trans-ancestry) populations. Details and results of published MR studies included in the meta-analyses as well as results from de novo MR analyses of FinnGen data are provided in Additional file 1: Table S2.

### Diabetes mellitus

Genetically predicted higher BMI was associated with an increased risk of type 1 diabetes mellitus in UK Biobank but not in the FinnGen consortium, with high heterogeneity between studies (Additional file 1: Table S2). On the other hand, there was a consistent association of genetically predicted adulthood BMI with type 2 diabetes, with a combined *OR* of 2.03 (*95% CI* 1.88–2.19) (Additional file 1: Table S2).

### Diseases of the circulatory system

**Fig. 2:**
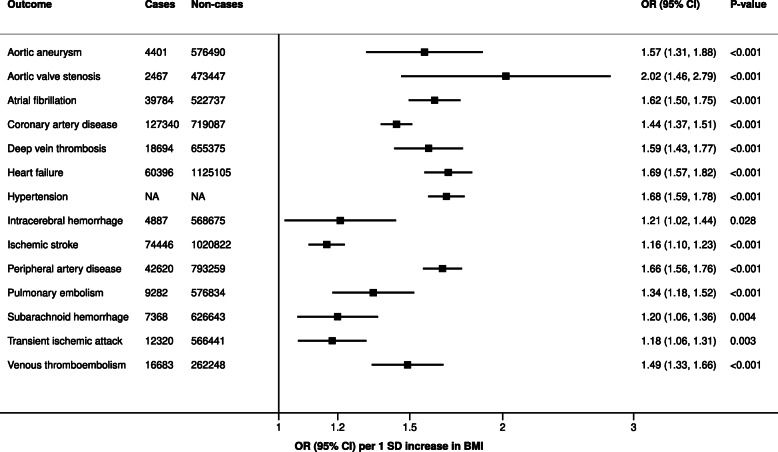
Meta-analysis results for genetically predicted BMI in relation to diseases of the circulatory system. Results are scaled per 1 *SD* increase of BMI. Analyses of coronary artery disease and peripheral artery disease include individuals of both European (the vast majority) and non-European ancestry; analyses of other outcomes include individuals of European ancestry only

Genetically predicted higher BMI was associated with increased risk of all 14 studied diseases of the circulatory system (Fig. 2, Additional file 1: Table S2). The strongest associations were for aortic valve stenosis (*OR* 2.02, *95% CI* 1.46–2.79), followed by heart failure (*OR* 1.69, *95% CI* 1.57–1.82) and hypertension (*OR* 1.68, *95% CI* 1.59–1.78). The associations were weaker for all stroke types, with *OR*s ranging from 1.16 (*95% CI* 1.10–1.23) for ischemic stroke to 1.21 (*95% CI* 1.02–1.44) for intracerebral hemorrhage. Excluding the study based on a Chinese population had a minor impact on the results for peripheral artery disease (*OR* 1.65, *95% CI* 1.55–1.75). High heterogeneity between studies was only observed in the analyses of aortic valve stenosis, atrial fibrillation, and hypertension, but this was due to different magnitude of positive associations rather than a lack of association in one of the studies (Additional file 1: Table S2).

### Diseases of the respiratory system

MR studies on diseases of the respiratory system were scarce, with results reported only for asthma and chronic obstructive pulmonary disease (COPD) mortality (Additional file 1: Table S2). In a meta-analysis of available independent study samples, the *OR* was 1.36 (*95% CI* 1.29–1.43) for asthma and 1.65 (*95% CI* 1.47–1.85) for COPD, with no heterogeneity among studies.

### Diseases of the digestive system

**Fig. 3:**
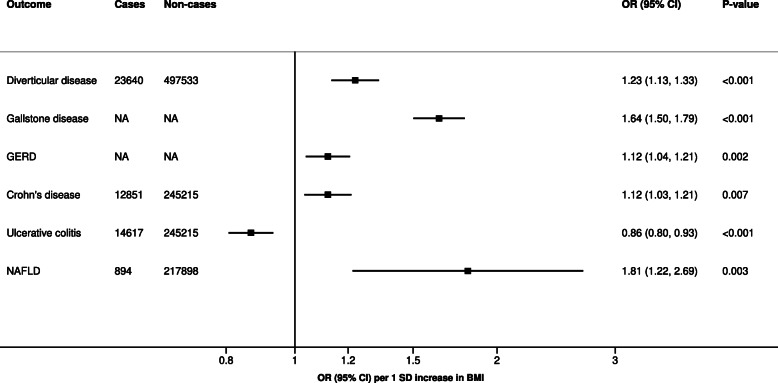
Meta-analysis results for genetically predicted BMI in relation to diseases of the digestive system. Results are scaled per 1 *SD* increase of BMI. All analyses include individuals of European ancestry only. GERD gastroesophageal reflux disease, NA not available, NAFLD nonalcoholic fatty liver disease

Genetically predicted higher BMI was associated with an increased risk of diverticular disease, gallstone disease, gastroesophageal reflux disease, Crohn’s disease, and nonalcoholic fatty liver disease, but with a lower risk of ulcerative colitis (Fig. 3, Additional file 1: Table S2). The strongest association was for nonalcoholic fatty liver disease (*OR* 1.81, *95% CI* 1.22–2.69). Results for gallstone disease remained essentially unchanged after removing the study based on a Chinese population (Additional file 1: Table S2). Moderate heterogeneity between studies was only observed in the analysis of gastroesophageal reflux disease.

### Diseases of the musculoskeletal system

Published MR studies of BMI and diseases of the musculoskeletal diseases were available for Dupuytren’s disease, gout, osteoarthritis, and rheumatoid arthritis (Additional file 1: Table S2). Higher genetically predicted BMI was associated with a decreased risk of Dupuytren’s disease but with an increased risk of the other three musculoskeletal diseases. The combined *OR*s were 0.77 (*95% CI* 0.69–0.87) for Dupuytren’s disease, 1.92 (*95% CI* 1.60–2.30) for gout, 1.55 (*95% CI* 1.43–1.69) for osteoarthritis, and 1.27 (*95% CI* 1.17–1.39) for rheumatoid arthritis. There was high heterogeneity between studies on Dupuytren’s disease and moderate heterogeneity between studies on gout and rheumatoid arthritis. MR analysis of osteoporosis in the FinnGen consortium showed an *OR* of 0.81 (*95% CI* 0.65–0.99) per 1 *SD* increase in genetically predicted BMI (Additional file 1: Table S2).

### Diseases of the nervous system

Genetically predicted BMI was associated with multiple sclerosis (*OR* 1.26, *95% CI* 1.14–1.39) but not Alzheimer’s disease or amyotrophic lateral sclerosis (Additional file 1: Table S2). There was heterogeneity between estimates for Parkinson’s disease, with an inverse association of genetically predicted BMI and Parkinson’s disease found in the FinnGen consortium (*OR* 0.76, *95% CI* 0.60–0.96) but not in the Parkinson’s disease genome-wide association study (*OR* 0.96, *95% CI* 0.83–1.12). The combined *OR* of Parkinson’s disease was 0.90 (*95% CI* 0.79–1.02).

### Neoplasms

**Fig. 4:**
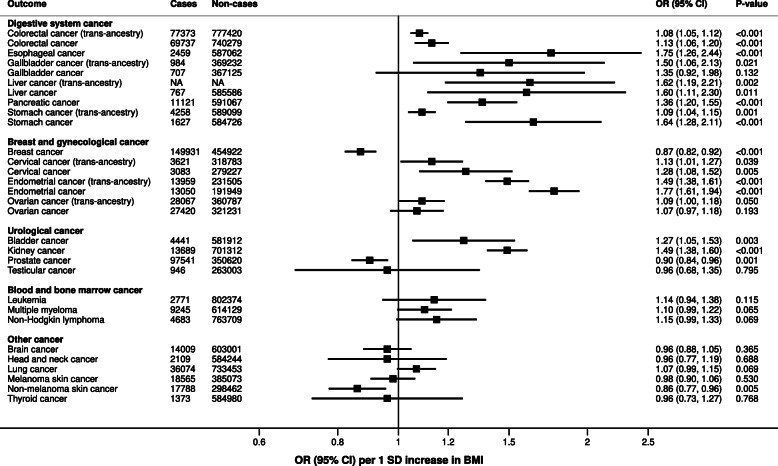
Meta-analysis results for genetically predicted BMI in relation to neoplasms. Results are scaled per 1 *SD* increase of BMI. Analyses include individuals of European ancestry only if not otherwise indicated (i.e., trans-ancestry). NA not available

Meta-analysis results showed that genetically predicted BMI was associated with an increased risk of cancers of the digestive system (i.e., esophageal, stomach, colorectal, pancreatic, liver, and gallbladder/biliary tract cancer), uterus (endometrial and cervical cancer), ovary, kidney, and bladder, but with a decreased risk of breast, prostate, and non-melanoma skin cancer (Fig. 4). There was no consistent and overall association with other cancers (Fig. 3, Additional file 1: Table S2). Results remained except for ovarian cancer when confining the study populations to individuals of European ancestry, but the magnitude of the association became weaker for colorectal cancer and stronger for stomach, endometrial, and cervical cancer (Additional file 1: Table S2).

## Discussion

This contemporary meta-analysis of MR studies of genetically predicted BMI in relation to 56 chronic diseases provides evidence in support of causal associations of excess adiposity with increased risk of type 2 diabetes mellitus, 14 circulatory system diseases, asthma, chronic obstructive pulmonary disease, five digestive system diseases, three musculoskeletal system diseases, multiple sclerosis, and cancers of the digestive system (six cancer sites), uterus, kidney, and bladder. In contrast, MR evidence indicates that high BMI is associated with a decreased risk of breast, prostate, and non-melanoma skin cancer, Dupuytren’s disease, and osteoporosis, and not likely associated with risk of Alzheimer disease, amyotrophic lateral sclerosis, or Parkinson’s disease.

This meta-analysis of MR studies found consistent associations of higher genetically predicted BMI and increased risk of type 2 diabetes mellitus and cardiovascular diseases. However, conflicting results were found for adulthood BMI in relation to type 1 diabetes mellitus. The inconsistent results might be related to different ages at onset of type 1 diabetes mellitus in the UK Biobank and FinnGen populations or that genetic instruments for adulthood BMI rather than childhood BMI were used. An MR study of genetically predicted childhood BMI showed a positive association with childhood-onset (<17 years) type 1 diabetes mellitus (*OR* 1.32, *95% CI* 1.06–1.64 per *SD* score increase in BMI based on 32 SNPs) [62]. Excess adiposity may increase the risk of type 2 diabetes mellitus and cardiovascular diseases by increasing fasting glucose, insulin, and triglyceride levels; raising blood pressure; and promoting systemic inflammation [63–65]. An MR study based on consortia data found that the genetic association of BMI with risk of coronary artery disease, peripheral artery disease, and stroke was partly mediated by systolic blood pressure and type 2 diabetes mellitus, but not materially mediated by lipids or smoking [19].

Higher genetically predicted BMI was associated with increased risk of several diseases of the respiratory, digestive, and musculoskeletal systems, including asthma, gallbladder disease, diverticular disease, nonalcoholic fatty liver disease, gout, osteoarthritis, and rheumatoid arthritis. The associations may be related to an obesity-related reduction in lung volume (for asthma), joint loading (for osteoarthritis), and alterations in microbiota composition, inflammatory mediators, and hormone levels. In contrast, higher genetically predicted BMI was associated with a modest lower risk of osteoporosis, possibly explained by mechanical stresses mediated through gravitational action. This finding confirms previous MR studies that have shown a positive association between genetically predicted BMI and bone mineral density [66, 67]. Genetically predicted BMI was also inversely associated with the risk of Dupuytren’s disease in an MR study based on data from a genome-wide association study on this outcome [30]. The mechanism behind this association is unclear but might be related to lower testosterone levels with increasing BMI [30]. For the two inflammatory bowel diseases, the direction of the association with genetically predicted BMI differed for Crohn’s disease (positive association) and ulcerative colitis (inverse association). A previous meta-analysis of observational studies found that BMI was positively associated with the risk of Crohn’s disease but unrelated to ulcerative colitis [68]. Hence, the observed inverse association between genetically predicted BMI and ulcerative colitis in the present meta-analysis of two MR studies may be a spurious finding. In fact, the inverse association was only significant in the IBD consortium but not in the FinnGen consortium.

This meta-analysis of MR studies provided evidence that excess adiposity increases the risk of multiple sclerosis but not Alzheimer’s disease, amyotrophic lateral sclerosis, or Parkinson’s disease. If anything, a suggestive inverse association was observed between genetically predicted BMI and Parkinson’s disease. This finding is consistent with the results of a previous meta-analysis that found that being underweight was associated with an increased risk of Parkinson’s disease [69].

The opposite direction of the associations of genetically predicted BMI with different cancers suggests different causal pathways for BMI and various cancers. The increased risk of cancers of the digestive system, uterus, kidney, and bladder may be mediated by alterations in insulin signaling, growth factors, adipose tissue-derived inflammation, and hormone levels. Higher genetically predicted BMI has been shown to relate to lower serum testosterone levels [70], and testosterone levels are positively associated with risk of breast, prostate, and skin cancer [71, 72]. Thus, the observed inverse associations of genetically predicted BMI with these cancers might, at least in part, be explained by lower testosterone levels in overweight and obese individuals. In premenopausal women, high BMI may lower breast cancer risk via decreased estradiol levels [73].

Heterogeneity was observed between estimates from individual studies in analyses of genetically predicted BMI and several disease outcomes (e.g., diabetes, aortic valve stenosis, atrial fibrillation, hypertension, and stomach, endometrial, bladder, head and neck, and lung cancer). The detected heterogeneity was mainly caused by the different magnitude of the associations across studies and may be related to different genetic instruments used or to different study populations with different characteristics.

A strength of MR studies is that confounding and reverse causation bias are reduced as BMI is proxied by genetic variants that generally are not related to self-selected behaviors and environmental exposures and are not modified by disease development. The validity of MR findings relies on the absence of pleiotropy (i.e., where a genetic variant is associated with more than one phenotype). Researchers of most MR studies included in the meta-analysis performed sensitivity analyses and found limited evidence that the associations were biased by pleiotropy. Another limitation in MR studies of obesity and other harmful exposures in relation to late-onset diseases is competing risk bias, which is a potential type of survival bias. It cannot be ruled out that this bias might have affected the results in some of the studies. A further shortcoming is that most MR studies comprised individuals of European ancestry and therefore cannot infer causality of the role of excess adiposity in chronic diseases in non-European populations.

## Conclusions

The totality of the evidence from published and de novo Mendelian randomization analyses supports a causal role of excess adiposity in a plurality of chronic diseases. Hence, continued efforts to reduce the prevalence of overweight and obesity are a major public health goal.

## Supplementary Information

**Additional file 1: Tables S1-S2**. Table S1 – Study quality assessment. Table S2 – Mendelian randomization studies included in the meta-analyses of genetically predicted body mass index in relation to diabetes mellitus, diseases of the circulatory, respiratory, digestive, musculoskeletal, and nervous systems, and neoplasms.

## Data Availability

All the data supporting the conclusions of this article are included within the article and its supplementary files.

## Abbreviations

BMI: Body mass index
*CI*: Confidence interval
MR: Mendelian randomization
*OR*: Odds ratio
*SD*: Standard deviation
SNPs: Single-nucleotide polymorphisms

## References

[1] Renehan AG, Tyson M, Egger M, Heller RF, Zwahlen M (2008). Body-mass index and incidence of cancer: a systematic review and meta-analysis of prospective observational studies. Lancet.

[2] Guh DP, Zhang W, Bansback N, Amarsi Z, Birmingham CL, Anis AH (2009). The incidence of co-morbidities related to obesity and overweight: a systematic review and meta-analysis. BMC Public Health.

[3] WHO Consultation on Obesity (1999: Geneva, Switzerland) & World Health Organization. Obesity: preventing and managing the global epidemic: report of a WHO consultation. Geneva, Switzerland: World Health Organization; 2000. https://apps.who.int/iris/handle/10665/42330. Accessed 18 Aug 2021. 11234459

[4] Perk J, De Backer G, Gohlke H, Graham I, Reiner Z, Verschuren M, Albus C, Benlian P, Boysen G, Cifkova R et al: European Guidelines on cardiovascular disease prevention in clinical practice (version 2012). The Fifth Joint Task Force of the European Society of Cardiology and Other Societies on Cardiovascular Disease Prevention in Clinical Practice (constituted by representatives of nine societies and by invited experts). Eur Heart J, 33(13):1635-1701. 10.1093/eurheartj/ehs092 22555213

[5] World Cancer Research Fund/American Institute for Cancer Research. Diet, nutrition, physical activity and cancer: a global perspective. In: The Third Expert Report: World Cancer Research Fund International; 2018. Available at http://dietandcancerreport.org/:. Accessed 18 Aug 2021.

[6] Choi EK, Park HB, Lee KH, Park JH, Eisenhut M, van der Vliet HJ, Kim G, Shin JI (2018). Body mass index and 20 specific cancers: re-analyses of dose-response meta-analyses of observational studies. Ann Oncol.

[7] Yuan S, Larsson SC (2020). An atlas on risk factors for type 2 diabetes: a wide-angled Mendelian randomisation study. Diabetologia.

[8] Hypponen E, Mulugeta A, Zhou A, Santhanakrishnan VK (2019). A data-driven approach for studying the role of body mass in multiple diseases: a phenome-wide registry-based case-control study in the UK Biobank. Lancet Digit Health.

[9] Nordestgaard BG, Palmer TM, Benn M, Zacho J, Tybjaerg-Hansen A, Davey Smith G, Timpson NJ (2012). The effect of elevated body mass index on ischemic heart disease risk: causal estimates from a Mendelian randomisation approach. PLoS Med.

[10] Klovaite J, Benn M, Nordestgaard BG (2015). Obesity as a causal risk factor for deep venous thrombosis: a Mendelian randomization study. J Intern Med.

[11] Huang Y, Xu M, Xie L, Wang T, Huang X, Lv X, Chen Y, Ding L, Lin L, Wang W, Bi Y, Sun Y, Zhang Y, Ning G (2016). Obesity and peripheral arterial disease: a Mendelian randomization analysis. Atherosclerosis.

[12] Chatterjee NA, Giulianini F, Geelhoed B, Lunetta KL, Misialek JR, Niemeijer MN, Rienstra M, Rose LM, Smith AV, Arking DE, Ellinor PT, Heeringa J, Lin H, Lubitz SA, Soliman EZ, Verweij N, Alonso A, Benjamin EJ, Gudnason V, Stricker BHC, van der Harst P, Chasman DI, Albert CM (2017). Genetic obesity and the risk of atrial fibrillation: causal estimates from Mendelian randomization. Circulation.

[13] van ‘t Hof FN, Vaucher J, Holmes MV, de Wilde A, Baas AF, Blankensteijn JD, Hofman A, Kiemeney LA, Rivadeneira F, Uitterlinden AG (2017). Genetic variants associated with type 2 diabetes and adiposity and risk of intracranial and abdominal aortic aneurysms. Eur J Hum Genet.

[14] Lindstrom S, Germain M, Crous-Bou M, Smith EN, Morange PE, van Hylckama VA, de Haan HG, Chasman D, Ridker P, Brody J (2017). Assessing the causal relationship between obesity and venous thromboembolism through a Mendelian Randomization study. Hum Genet.

[15] Larsson SC, Bäck M, Rees JMB, Mason AM, Burgess S (2019). Body mass index and body composition in relation to 14 cardiovascular conditions in UK Biobank: a Mendelian randomization study. Eur Heart J.

[16] Shah S, Henry A, Roselli C, Lin H, Sveinbjornsson G, Fatemifar G, Hedman AK, Wilk JB, Morley MP, Chaffin MD (2020). Genome-wide association and Mendelian randomisation analysis provide insights into the pathogenesis of heart failure. Nat Commun.

[17] Kaltoft M, Langsted A, Nordestgaard BG (2020). Obesity as a causal risk factor for aortic valve stenosis. J Am Coll Cardiol.

[18] Harshfield EL, Georgakis MK, Malik R, Dichgans M, Markus HS (2021). Modifiable lifestyle factors and risk of stroke: a Mendelian randomization analysis. Stroke.

[19] Gill D, Zuber V, Dawson J, Pearson-Stuttard J, Carter AR, Sanderson E, Karhunen V, Levin MG, Wootton RE, Klarin D, Tsao PS, Tsilidis KK, Damrauer SM, Burgess S, Elliott P (2021). Risk factors mediating the effect of body mass index and waist-to-hip ratio on cardiovascular outcomes: Mendelian randomization analysis. Int J Obes (Lond).

[20] Giontella A, Lotta LA, Overton JD, Baras A, Regeneron Genetics C, Minuz P, Melander O, Gill D, Fava C (2021). Causal effect of adiposity measures on blood pressure traits in 2 urban Swedish cohorts: a Mendelian randomization study. J Am Heart Assoc.

[21] Karhunen V, Bakker MK, Ruigrok YM, Gill D, Larsson SC. Modifiable Risk Factors for Intracranial Aneurysm and Aneurysmal Subarachnoid Hemorrhage: A Mendelian Randomization Study. J Am Heart Assoc. 2021;10(22):e022277. 10.1161/JAHA.121.022277. 10.1161/JAHA.121.022277 PMC8751955 34729997

[22] Sun YQ, Brumpton BM, Langhammer A, Chen Y, Kvaloy K, Mai XM (2020). Adiposity and asthma in adults: a bidirectional Mendelian randomisation analysis of The HUNT Study. Thorax.

[23] Au Yeung SL, Li AM, Schooling CM (2021). A life course approach to elucidate the role of adiposity in asthma risk: evidence from a Mendelian randomisation study. J Epidemiol Community Health.

[24] Stender S, Nordestgaard BG, Tybjaerg-Hansen A (2013). Elevated body mass index as a causal risk factor for symptomatic gallstone disease: a Mendelian randomization study. Hepatology.

[25] Pang Y, Kartsonaki C, Lv J, Millwood IY, Yu C, Guo Y, Chen Y, Bian Z, Yang L, Chen J, Clarke R, Walters R, Wu S, Li H, Holmes MV, Li L, Chen Z (2020). Observational and genetic associations of body mass index and hepatobiliary diseases in a relatively lean Chinese population. JAMA Netw Open.

[26] Carreras-Torres R, Ibanez-Sanz G, Obon-Santacana M, Duell EJ, Moreno V (2020). Identifying environmental risk factors for inflammatory bowel diseases: a Mendelian randomization study. Sci Rep.

[27] Yuan S, Gill D, Giovannucci EL, Larsson SC (2021). Obesity, type 2 diabetes, lifestyle factors, and risk of gallstone disease: a Mendelian randomization investigation. Clin Gastroenterol Hepatol.

[28] Yuan S, Larsson SC (2021). Genetically predicted adiposity, diabetes, and lifestyle factors in relation to diverticular disease. Clin Gastroenterol Hepatol.

[29] Green HD, Beaumont RN, Wood AR, Hamilton B, Jones SE, Goodhand JR, Kennedy NA, Ahmad T, Yaghootkar H, Weedon MN, Frayling TM, Tyrrell J (2020). Genetic evidence that higher central adiposity causes gastro-oesophageal reflux disease: a Mendelian randomization study. Int J Epidemiol.

[30] Majeed M, Wiberg A, Ng M, Holmes MV, Furniss D (2021). The relationship between body mass index and the risk of development of Dupuytren’s disease: a Mendelian randomization study. J Hand Surg Eur Vol.

[31] Larsson SC, Burgess S, Michaelsson K (2018). Genetic association between adiposity and gout: a Mendelian randomization study. Rheumatology (Oxford).

[32] Hindy G, Akesson KE, Melander O, Aragam KG, Haas ME, Nilsson PM, Kadam UT, Orho-Melander M (2019). Cardiometabolic polygenic risk scores and osteoarthritis outcomes: a Mendelian randomization study using data from the Malmo Diet and Cancer Study and the UK Biobank. Arthritis Rheumatol.

[33] Funck-Brentano T, Nethander M, Moverare-Skrtic S, Richette P, Ohlsson C (2019). Causal factors for knee, hip, and hand osteoarthritis: a Mendelian randomization study in the UK Biobank. Arthritis Rheumatol.

[34] Tang B, Shi H, Alfredsson L, Klareskog L, Padyukov L, Jiang X (2021). Obesity-related traits and the development of rheumatoid arthritis: evidence from genetic data. Arthritis Rheumatol.

[35] Nordestgaard LT, Tybjaerg-Hansen A, Nordestgaard BG, Frikke-Schmidt R (2017). Body mass index and risk of Alzheimer’s disease: a Mendelian randomization study of 399,536 individuals. J Clin Endocrinol Metab.

[36] Larsson SC, Traylor M, Malik R, Dichgans M, Burgess S, Markus HS (2017). CoStream consortium obotIGoAsP: modifiable pathways in Alzheimer’s disease: Mendelian randomisation analysis. BMJ.

[37] Zeng P, Yu X, Xu H (2019). Association between premorbid body mass index and amyotrophic lateral sclerosis: causal inference through genetic approaches. Front Neurol.

[38] Yuan S, Xiong Y, Larsson SC (2020). An atlas on risk factors for multiple sclerosis: a Mendelian randomization study. J Neurol.

[39] Noyce AJ, Bandres-Ciga S, Kim J, Heilbron K, Kia D, Hemani G, Xue A, Lawlor DA, Smith GD, Duran R, Gan-Or Z, Blauwendraat C, Gibbs JR, Hinds DA, Yang J, Visscher P, Cuzick J, Morris H, Hardy J, Wood NW, Nalls MA, Singleton AB, 23andMe Research Team, International Parkinson's Disease Genomics Consortium (IPDGC) (2019). The Parkinson’s disease Mendelian randomization research portal. Mov Disord.

[40] Thrift AP, Shaheen NJ, Gammon MD, Bernstein L, Reid BJ, Onstad L, et al. Obesity and risk of esophageal adenocarcinoma and Barrett’s esophagus: a Mendelian randomization study. J Natl Cancer Inst. 2014;106(11). 10.1093/jnci/dju252. 10.1093/jnci/dju252 PMC4200028 25269698

[41] Benn M, Tybjaerg-Hansen A, Smith GD, Nordestgaard BG (2016). High body mass index and cancer risk-a Mendelian randomisation study. Eur J Epidemiol.

[42] Mao Y, Yan C, Lu Q, Zhu M, Yu F, Wang C, Dai J, Ma H, Hu Z, Shen H, Jin G (2017). Genetically predicted high body mass index is associated with increased gastric cancer risk. Eur J Hum Genet.

[43] Johansson M, Carreras-Torres R, Scelo G, Purdue MP, Mariosa D, Muller DC, Timpson NJ, Haycock PC, Brown KM, Wang Z, Ye Y, Hofmann JN, Foll M, Gaborieau V, Machiela MJ, Colli LM, Li P, Garnier JG, Blanche H, Boland A, Burdette L, Prokhortchouk E, Skryabin KG, Yeager M, Radojevic-Skodric S, Ognjanovic S, Foretova L, Holcatova I, Janout V, Mates D, Mukeriya A, Rascu S, Zaridze D, Bencko V, Cybulski C, Fabianova E, Jinga V, Lissowska J, Lubinski J, Navratilova M, Rudnai P, Benhamou S, Cancel-Tassin G, Cussenot O, Weiderpass E, Ljungberg B, Tumkur Sitaram R, Häggström C, Bruinsma F, Jordan SJ, Severi G, Winship I, Hveem K, Vatten LJ, Fletcher T, Larsson SC, Wolk A, Banks RE, Selby PJ, Easton DF, Andreotti G, Beane Freeman LE, Koutros S, Männistö S, Weinstein S, Clark PE, Edwards TL, Lipworth L, Gapstur SM, Stevens VL, Carol H, Freedman ML, Pomerantz MM, Cho E, Wilson KM, Gaziano JM, Sesso HD, Freedman ND, Parker AS, Eckel-Passow JE, Huang WY, Kahnoski RJ, Lane BR, Noyes SL, Petillo D, Teh BT, Peters U, White E, Anderson GL, Johnson L, Luo J, Buring J, Lee IM, Chow WH, Moore LE, Eisen T, Henrion M, Larkin J, Barman P, Leibovich BC, Choueiri TK, Lathrop GM, Deleuze JF, Gunter M, McKay JD, Wu X, Houlston RS, Chanock SJ, Relton C, Richards JB, Martin RM, Davey Smith G, Brennan P (2019). The influence of obesity-related factors in the etiology of renal cell carcinoma-a Mendelian randomization study. PLoS Med.

[44] Saunders CN, Cornish AJ, Kinnersley B, Law PJ, Claus EB, Il'yasova D, Schildkraut J, Barnholtz-Sloan JS, Olson SH, Bernstein JL, Lai RK, Chanock S, Rajaraman P, Johansen C, Jenkins RB, Melin BS, Wrensch MR, Sanson M, Bondy ML, Houlston RS (2020). Lack of association between modifiable exposures and glioma risk: a Mendelian randomization analysis. Neuro Oncol.

[45] Lu Y, Gentiluomo M, Lorenzo-Bermejo J, Morelli L, Obazee O, Campa D, Canzian F (2020). Mendelian randomisation study of the effects of known and putative risk factors on pancreatic cancer. J Med Genet.

[46] Dusingize JC, Olsen CM, An J, Pandeya N, Law MH, Thompson BS, Goldstein AM, Iles MM, Webb PM, Neale RE, Ong JS, MacGregor S, Whiteman DC (2020). Body mass index and height and risk of cutaneous melanoma: Mendelian randomization analyses. Int J Epidemiol.

[47] Went M, Cornish AJ, Law PJ, Kinnersley B, van Duin M, Weinhold N, Forsti A, Hansson M, Sonneveld P, Goldschmidt H (2020). Search for multiple myeloma risk factors using Mendelian randomization. Blood Adv.

[48] Masuda T, Ogawa K, Kamatani Y, Murakami Y, Kimura T, Okada Y (2020). A Mendelian randomization study identified obesity as a causal risk factor of uterine endometrial cancer in Japanese. Cancer Sci.

[49] Bull CJ, Bell JA, Murphy N, Sanderson E, Davey Smith G, Timpson NJ, Banbury BL, Albanes D, Berndt SI, Bezieau S (2020). Adiposity, metabolites, and colorectal cancer risk: Mendelian randomization study. BMC Med.

[50] Suzuki S, Goto A, Nakatochi M, Narita A, Yamaji T, Sawada N, Katagiri R, Iwagami M, Hanyuda A, Hachiya T, Sutoh Y, Oze I, Koyanagi YN, Kasugai Y, Taniyama Y, Ito H, Ikezaki H, Nishida Y, Tamura T, Mikami H, Takezaki T, Suzuki S, Ozaki E, Kuriki K, Takashima N, Arisawa K, Takeuchi K, Tanno K, Shimizu A, Tamiya G, Hozawa A, Kinoshita K, Wakai K, Sasaki M, Yamamoto M, Matsuo K, Tsugane S, Iwasaki M (2021). Body mass index and colorectal cancer risk: a Mendelian randomization study. Cancer Sci.

[51] Vithayathil M, Carter P, Kar S, Mason AM, Burgess S, Larsson SC (2021). Body size and composition and risk of site-specific cancers in the UK Biobank and large international consortia: a Mendelian randomisation study. PLoS Med.

[52] Zhou W, Liu G, Hung RJ, Haycock PC, Aldrich MC, Andrew AS, Arnold SM, Bickeboller H, Bojesen SE, Brennan P (2021). Causal relationships between body mass index, smoking and lung cancer: univariable and multivariable Mendelian randomization. Int J Cancer.

[53] Barahona Ponce C, Scherer D, Brinster R, Boekstegers F, Marcelain K, Garate-Calderon V, Muller B, de Toro G, Retamales J, Barajas O (2021). Gallstones, body mass index, C-reactive protein, and gallbladder cancer: Mendelian randomization analysis of Chilean and European Genotype Data. Hepatology.

[54] Davey Smith G, Ebrahim S (2003). Mendelian randomization: can genetic epidemiology contribute to understanding environmental determinants of disease?. Int J Epidemiol.

[55] Smith GD, Davies NM, Egger M, Gallo V, Golub R, Higgins JP, Langenberg C, Loder EW, Richards JB, Richmond RC (2019). STROBE-MR: guidelines for strengthening the reporting of Mendelian randomization studies. PeerJ Preprints.

[56] Burgess S, Davey Smith G, Davies NM, Dudbridge F, Gill D, Glymour MM, Hartwig FP, Holmes MV, Minelli C, Relton CL, Theodoratou E (2019). Guidelines for performing Mendelian randomization investigations. Wellcome Open Res.

[57] FinnGen consortium. FinnGen Documentation of R5 release, 2021. Web: https://finngen.gitbook.io/documentation/ [access date 12 Aug 2021].

[58] Pulit SL, Stoneman C, Morris AP, Wood AR, Glastonbury CA, Tyrrell J, Yengo L, Ferreira T, Marouli E, Ji Y, Yang J, Jones S, Beaumont R, Croteau-Chonka DC, Winkler TW, Hattersley AT, Loos RJF, Hirschhorn JN, Visscher PM, Frayling TM, Yaghootkar H, Lindgren CM, GIANT Consortium (2019). Meta-analysis of genome-wide association studies for body fat distribution in 694 649 individuals of European ancestry. Hum Mol Genet.

[59] Higgins JP, Thompson SG (2002). Quantifying heterogeneity in a meta-analysis. Stat Med.

[60] Locke AE, Kahali B, Berndt SI, Justice AE, Pers TH, Day FR, Powell C, Vedantam S, Buchkovich ML, Yang J (2015). Genetic studies of body mass index yield new insights for obesity biology. Nature.

[61] Yengo L, Sidorenko J, Kemper KE, Zheng Z, Wood AR, Weedon MN, Frayling TM, Hirschhorn J, Yang J, Visscher PM (2018). Meta-analysis of genome-wide association studies for height and body mass index in approximately 700000 individuals of European ancestry. Hum Mol Genet.

[62] Censin JC, Nowak C, Cooper N, Bergsten P, Todd JA, Fall T (2017). Childhood adiposity and risk of type 1 diabetes: a Mendelian randomization study. PLoS Med.

[63] Fall T, Hagg S, Ploner A, Magi R, Fischer K, Draisma HH, Sarin AP, Benyamin B, Ladenvall C, Akerlund M (2015). Age- and sex-specific causal effects of adiposity on cardiovascular risk factors. Diabetes.

[64] Holmes MV, Lange LA, Palmer T, Lanktree MB, North KE, Almoguera B, Buxbaum S, Chandrupatla HR, Elbers CC, Guo Y, Hoogeveen RC, Li J, Li YR, Swerdlow DI, Cushman M, Price TS, Curtis SP, Fornage M, Hakonarson H, Patel SR, Redline S, Siscovick DS, Tsai MY, Wilson JG, van der Schouw YT, FitzGerald GA, Hingorani AD, Casas JP, de Bakker PIW, Rich SS, Schadt EE, Asselbergs FW, Reiner AP, Keating BJ (2014). Causal effects of body mass index on cardiometabolic traits and events: a Mendelian randomization analysis. Am J Hum Genet.

[65] Emdin CA, Khera AV, Natarajan P, Klarin D, Zekavat SM, Hsiao AJ, Kathiresan S (2017). Genetic association of waist-to-hip ratio with cardiometabolic traits, type 2 diabetes, and coronary heart disease. JAMA.

[66] Song J, Zhang R, Lv L, Liang J, Wang W, Liu R, Dang X (2020). The relationship between body mass index and bone mineral density: a Mendelian randomization study. Calcif Tissue Int.

[67] Ma B, Li C, Pan J, Zhang S, Dong H, Wu Y, Lv J (2021). Causal associations of anthropometric measurements with fracture risk and bone mineral density: a Mendelian randomization study. J Bone Miner Res.

[68] Rahmani J, Kord-Varkaneh H, Hekmatdoost A, Thompson J, Clark C, Salehisahlabadi A, Day AS, Jacobson K (2019). Body mass index and risk of inflammatory bowel disease: a systematic review and dose-response meta-analysis of cohort studies of over a million participants. Obes Rev.

[69] Rahmani J, Roudsari AH, Bawadi H, Clark C, Ryan PM, Salehisahlabadi A, et al. Body mass index and risk of Parkinson, Alzheimer, dementia, and dementia mortality: a systematic review and dose-response meta-analysis of cohort studies among 5 million participants. Nutr Neurosci. 2020:1–9. 10.1080/1028415X.2020.1758888. 10.1080/1028415X.2020.1758888 33107811

[70] Eriksson J, Haring R, Grarup N, Vandenput L, Wallaschofski H, Lorentzen E, Hansen T, Mellstrom D, Pedersen O, Nauck M (2017). Causal relationship between obesity and serum testosterone status in men: a bi-directional Mendelian randomization analysis. PLoS One.

[71] Ruth KS, Day FR, Tyrrell J, Thompson DJ, Wood AR, Mahajan A, Beaumont RN, Wittemans L, Martin S, Busch AS (2020). Using human genetics to understand the disease impacts of testosterone in men and women. Nat Med.

[72] Watts EL, Perez-Cornago A, Knuppel A, Tsilidis KK, Key TJ, Travis RC (2021). Prospective analyses of testosterone and sex hormone-binding globulin with the risk of 19 types of cancer in men and postmenopausal women in UK Biobank. Int J Cancer.

[73] Garcia-Estevez L, Cortes J, Perez S, Calvo I, Gallegos I, Moreno-Bueno G (2021). Obesity and breast cancer: a paradoxical and controversial relationship influenced by menopausal status. Front Oncol.

