# Supplementary material for: Causal role of high body mass index in multiple chronic diseases: a systematic review and meta-analysis of Mendelian randomization studies
Source: BMC Med. 2021 Dec 15;19:320. doi: 10.1186/s12916-021-02188-x (PMC8672504; doi:10.1186/s12916-021-02188-x)
Supplement: Supplementary file 1 — Additional file 1: Tables S1-S2. Table S1 – Study quality assessment. Table S2 – Mendelian randomization studies included in the meta-analyses of genetically predicted body mass index in relation to diabetes mellitus, diseases of the circulatory, respiratory, digestive, musculoskeletal, and nervous systems, and neoplasms. [file 12916_2021_2188_MOESM1_ESM.docx]

**Table S1.** Study quality assessment*

| **Reference** | **Disease** | **1. Title & abstract** | **2. Background & objective** | **3. Design & data sources** | **4. Study sample** | **5. Selection of genetic variants** | **6. Primary analysis** | **7. Sensitivity analyses** | **8. Software** | **9. Data presentation** | **10. Limitations, interpretation** |
| --- | --- | --- | --- | --- | --- | --- | --- | --- | --- | --- | --- |
| Hyppönen et al., 2019 | Type 1 diabetes mellitus | ¬ | ¬ | ¬ | ¬ | ¬ | ¬ | ¬ | ¬ | ¬ | ¬ |
| Yuan & Larsson, 2020 | Type 2 diabetes mellitus | ¬ | ¬ | ¬ | ¬ | ¬ | ¬ | ¬ | ¬ | ¬ | ¬ |
| van 't Hof, et al., 2017 | Aortic aneurysm | ¬ | ¬ | ¬ | ¬ | ¬ | ¬ | ¬ | – | ¬ | ¬ |
| Larsson et al., 2020 | Aortic aneurysm | ¬ | ¬ | ¬ | ¬ | ¬ | ¬ | ¬ | ¬ | ¬ | ¬ |
| Kaltoft et al., 2020 | Aortic valve stenosis | ¬ | ¬ | ¬ | ¬ | ¬ | ¬ | – | ¬ | ¬ | ¬ |
| Larsson et al., 2020 | Aortic valve stenosis | ¬ | ¬ | ¬ | ¬ | ¬ | ¬ | ¬ | ¬ | ¬ | ¬ |
| Chatterjee, et al., 2017 | Atrial fibrillation | ¬ | ¬ | ¬ | ¬ | ¬ | ¬ | ¬ | ¬ | ¬ | ¬ |
| Larsson et al., 2020 | Atrial fibrillation | ¬ | ¬ | ¬ | ¬ | ¬ | ¬ | ¬ | ¬ | ¬ | ¬ |
| Gill et al., 2021 | Coronary artery disease | ¬ | ¬ | ¬ | ¬ | – | ¬ | ¬ | ¬ | ¬ | ¬ |
| Nordestgaard et al 2012 | Coronary artery disease | ¬ | ¬ | ¬ | ¬ | ¬ | ¬ | – | ¬ | ¬ | ¬ |
| Larsson et al., 2020 | Coronary artery disease | ¬ | ¬ | ¬ | ¬ | ¬ | ¬ | ¬ | ¬ | ¬ | ¬ |
| Klovaite et al., 2015 | Deep vein thrombosis | ¬ | ¬ | ¬ | ¬ | ¬ | ¬ | ¬ | ¬ | ¬ | ¬ |
| Larsson et al., 2020 | Deep vein thrombosis | ¬ | ¬ | ¬ | ¬ | ¬ | ¬ | ¬ | ¬ | ¬ | ¬ |
| Shah et al., 2020 | Heart failure | ¬ | ¬ | ¬ | ¬ | ¬ | ¬ | ¬ | ¬ | ¬ | ¬ |
| Giontella et al., 2021 | Hypertension | ¬ | ¬ | ¬ | ¬ | ¬ | ¬ | ¬ | ¬ | ¬ | ¬ |
| Larsson et al., 2020 | Hypertension | ¬ | ¬ | ¬ | ¬ | ¬ | ¬ | ¬ | ¬ | ¬ | ¬ |
| Harshfield et al., 2021 | Intracerebral hemorrhage | ¬ | ¬ | ¬ | ¬ | ¬ | ¬ | ¬ | ¬ | ¬ | ¬ |
| Larsson et al., 2020 | Intracerebral hemorrhage | ¬ | ¬ | ¬ | ¬ | ¬ | ¬ | ¬ | ¬ | ¬ | ¬ |
| Harshfield et al., 2021 | Ischemic stroke | ¬ | ¬ | ¬ | ¬ | ¬ | ¬ | ¬ | ¬ | ¬ | ¬ |
| Larsson et al., 2020 | Ischemic stroke | ¬ | ¬ | ¬ | ¬ | ¬ | ¬ | ¬ | ¬ | ¬ | ¬ |
| Huang et al., 2016 | Peripheral artery disease | ¬ | ¬ | ¬ | ¬ | ¬ | ¬ | – | – | ¬ | ¬ |
| Gill et al., 2021 | Peripheral artery disease | ¬ | ¬ | ¬ | ¬ | – | ¬ | ¬ | ¬ | ¬ | ¬ |
| Larsson et al., 2020 | Peripheral artery disease | ¬ | ¬ | ¬ | ¬ | ¬ | ¬ | ¬ | ¬ | ¬ | ¬ |
| Larsson et al., 2020 | Pulmonary emobolism | ¬ | ¬ | ¬ | ¬ | ¬ | ¬ | ¬ | ¬ | ¬ | ¬ |
| Karhunen et al., 2021 | Subarachnoid hemorrhage | ¬ | ¬ | ¬ | ¬ | ¬ | ¬ | ¬ | – | ¬ | ¬ |
| Larsson et al., 2020 | Subarachnoid hemorrhage | ¬ | ¬ | ¬ | ¬ | ¬ | ¬ | ¬ | ¬ | ¬ | ¬ |
| Larsson et al., 2020 | Transient ischemic attack | ¬ | ¬ | ¬ | ¬ | ¬ | ¬ | ¬ | ¬ | ¬ | ¬ |
| Lindström et al., 2017 | Venous thromboembolism | ¬ | ¬ | ¬ | ¬ | ¬ | – | ¬ | – | ¬ | ¬ |
| Sun et al., 2020 | Asthma | ¬ | ¬ | ¬ | ¬ | ¬ | ¬ | ¬ | ¬ | ¬ | ¬ |
| Au Yeung et al., 2021 | Asthma | ¬ | ¬ | ¬ | ¬ | ¬ | ¬ | ¬ | ¬ | ¬ | ¬ |
| Hyppönen et al, 2019 | Chronic obstructive pulmonary disease | ¬ | ¬ | ¬ | ¬ | ¬ | ¬ | ¬ | ¬ | ¬ | ¬ |
| Yuan & Larsson., 2021 | Diverticular disease | ¬ | ¬ | ¬ | ¬ | ¬ | ¬ | ¬ | ¬ | ¬ | ¬ |
| Stender et al., 2013 | Gallstone disease | ¬ | ¬ | ¬ | ¬ | ¬ | ¬ | – | ¬ | ¬ | ¬ |
| Pang et al., 2020 | Gallstone disease | ¬ | ¬ | ¬ | – | ¬ | ¬ | – | ¬ | ¬ | ¬ |
| Yuan et al., 2021 | Gallstone disease | ¬ | ¬ | ¬ | ¬ | ¬ | ¬ | ¬ | ¬ | ¬ | ¬ |
| Green et al., 2020 | Gastroesophageal reflux disease | ¬ | ¬ | ¬ | – | ¬ | ¬ | ¬ | – | ¬ | ¬ |
| Carreras-T. et al., 2020 | Crohn’s disease and ulcerative colitis | ¬ | ¬ | ¬ | ¬ | ¬ | ¬ | ¬ | ¬ | ¬ | ¬ |
| Pang et al., 2020 | Nonalcoholic fatty liver disease | ¬ | ¬ | ¬ | – | ¬ | ¬ | – | ¬ | ¬ | ¬ |
| Majeed et al. 2021 | Dupuytren’s disease | ¬ | ¬ | ¬ | – | ¬ | ¬ | ¬ | ¬ | ¬ | ¬ |
| Larsson et al., 2018 | Gout | ¬ | ¬ | ¬ | ¬ | ¬ | ¬ | ¬ | ¬ | ¬ | ¬ |
| Hindy et al., 2019 | Osteoarthritis | ¬ | ¬ | ¬ | ¬ | ¬ | ¬ | ¬ | ¬ | ¬ | ¬ |
| Funck-B. et al., 2019 | Osteoarthritis | ¬ | ¬ | ¬ | ¬ | ¬ | ¬ | ¬ | ¬ | ¬ | ¬ |
| Tang et al., 2021 | Rheumatoid arthritis | ¬ | ¬ | ¬ | ¬ | ¬ | ¬ | ¬ | ¬ | ¬ | ¬ |
| Larsson et al., 2017 | Alzheimer’s disease | ¬ | ¬ | ¬ | ¬ | ¬ | ¬ | ¬ | ¬ | ¬ | ¬ |
| Nordestgaard et al 2017 | Alzheimer’s disease | ¬ | ¬ | ¬ | ¬ | ¬ | ¬ | – | ¬ | ¬ | ¬ |
| Zeng et al., 2019 | Amyotrophic lateral sclerosis | ¬ | ¬ | ¬ | ¬ | ¬ | ¬ | ¬ | ¬ | ¬ | ¬ |
| Yuan et al., 2021 | Multiple sclerosis | ¬ | ¬ | ¬ | ¬ | ¬ | ¬ | ¬ | ¬ | ¬ | ¬ |
| Noyce et al., 2019 | Parkinson’s disease | ¬ | ¬ | ¬ | ¬ | ¬ | ¬ | ¬ | ¬ | ¬ | ¬ |
| Bull et al., 2020 | Colorectal cancer | ¬ | ¬ | ¬ | ¬ | ¬ | ¬ | ¬ | ¬ | ¬ | ¬ |
| Benn et al., 2016 | Colorectal cancer | ¬ | ¬ | ¬ | ¬ | ¬ | ¬ | – | ¬ | ¬ | ¬ |
| Suzuki et al., 2021 | Colorectal cancer | ¬ | ¬ | ¬ | ¬ | ¬ | ¬ | ¬ | ¬ | ¬ | ¬ |
| Vithayathil et al., 2021 | Colorectal cancer | ¬ | ¬ | ¬ | ¬ | ¬ | ¬ | ¬ | ¬ | ¬ | ¬ |
| Thrift et al., 2012 | Esophageal cancer | ¬ | ¬ | ¬ | ¬ | ¬ | ¬ | – | ¬ | ¬ | ¬ |
| Vithayathil et al., 2021 | Esophageal cancer | ¬ | ¬ | ¬ | ¬ | ¬ | ¬ | ¬ | ¬ | ¬ | ¬ |
| Barahona-P. et al., 2020 | Gallbladder cancer | ¬ | ¬ | ¬ | ¬ | ¬ | ¬ | ¬ | ¬ | ¬ | ¬ |
| Vithayathil et al., 2021 | Gallbladder cancer | ¬ | ¬ | ¬ | ¬ | ¬ | ¬ | ¬ | ¬ | ¬ | ¬ |
| Lu et al., 2020 | Pancreatic cancer | ¬ | ¬ | ¬ | ¬ | ¬ | ¬ | ¬ | ¬ | ¬ | ¬ |
| Vithayathil et al., 2021 | Pancreatic cancer | ¬ | ¬ | ¬ | ¬ | ¬ | ¬ | ¬ | ¬ | ¬ | ¬ |
| Mao et al., 2017 | Stomach cancer | ¬ | ¬ | ¬ | ¬ | ¬ | ¬ | ¬ | ¬ | ¬ | ¬ |
| Vithayathil et al., 2021 | Stomach cancer | ¬ | ¬ | ¬ | ¬ | ¬ | ¬ | ¬ | ¬ | ¬ | ¬ |
| Benn et al., 2016 | Breast cancer | ¬ | ¬ | ¬ | ¬ | ¬ | ¬ | – | ¬ | ¬ | ¬ |
| Vithayathil et al., 2021 | Breast cancer | ¬ | ¬ | ¬ | ¬ | ¬ | ¬ | ¬ | ¬ | ¬ | ¬ |
| Mazuda et al. 2020 | Cervical cancer | ¬ | ¬ | ¬ | ¬ | ¬ | ¬ | ¬ | ¬ | ¬ | ¬ |
| Vithayathil et al., 2021 | Cervical cancer | ¬ | ¬ | ¬ | ¬ | ¬ | ¬ | ¬ | ¬ | ¬ | ¬ |
| Mazuda et al. 2020 | Endometrial cancer | ¬ | ¬ | ¬ | ¬ | ¬ | ¬ | ¬ | ¬ | ¬ | ¬ |
| Vithayathil et al., 2021 | Endometrial cancer | ¬ | ¬ | ¬ | ¬ | ¬ | ¬ | ¬ | ¬ | ¬ | ¬ |
| Mazuda et al. 2020 | Ovarian cancer | ¬ | ¬ | ¬ | ¬ | ¬ | ¬ | ¬ | ¬ | ¬ | ¬ |
| Vithayathil et al., 2021 | Ovarian cancer | ¬ | ¬ | ¬ | ¬ | ¬ | ¬ | ¬ | ¬ | ¬ | ¬ |
| Vithayathil et al., 2021 | Bladder cancer | ¬ | ¬ | ¬ | ¬ | ¬ | ¬ | ¬ | ¬ | ¬ | ¬ |
| Benn et al., 2016 | Kidney cancer | ¬ | ¬ | ¬ | ¬ | ¬ | ¬ | – | ¬ | ¬ | ¬ |
| Johansson et al., 2019 | Kidney cancer | ¬ | ¬ | ¬ | ¬ | ¬ | ¬ | ¬ | ¬ | ¬ | ¬ |
| Vithayathil et al., 2021 | Kidney cancer | ¬ | ¬ | ¬ | ¬ | ¬ | ¬ | ¬ | ¬ | ¬ | ¬ |
| Benn et al., 2016 | Prostate cancer | ¬ | ¬ | ¬ | ¬ | ¬ | ¬ | – | ¬ | ¬ | ¬ |
| Vithayathil et al., 2021 | Prostate cancer | ¬ | ¬ | ¬ | ¬ | ¬ | ¬ | ¬ | ¬ | ¬ | ¬ |
| Vithayathil et al., 2021 | Testicular cancer | ¬ | ¬ | ¬ | ¬ | ¬ | ¬ | ¬ | ¬ | ¬ | ¬ |
| Vithayathil et al., 2021 | Leukemia | ¬ | ¬ | ¬ | ¬ | ¬ | ¬ | ¬ | ¬ | ¬ | ¬ |
| Went et al., 2020 | Multiple myeloma | ¬ | ¬ | ¬ | ¬ | ¬ | ¬ | ¬ | ¬ | ¬ | ¬ |
| Vithayathil et al., 2021 | Multiple myeloma | ¬ | ¬ | ¬ | ¬ | ¬ | ¬ | ¬ | ¬ | ¬ | ¬ |
| Vithayathil et al., 2021 | Non-Hodgkin’s lymphoma | ¬ | ¬ | ¬ | ¬ | ¬ | ¬ | ¬ | ¬ | ¬ | ¬ |
| Saunders et al., 2020 | Brain cancer | ¬ | ¬ | ¬ | ¬ | ¬ | ¬ | ¬ | ¬ | ¬ | ¬ |
| Vithayathil et al., 2021 | Brain cancer | ¬ | ¬ | ¬ | ¬ | ¬ | ¬ | ¬ | ¬ | ¬ | ¬ |
| Vithayathil et al., 2021 | Head-neck cancer | ¬ | ¬ | ¬ | ¬ | ¬ | ¬ | ¬ | ¬ | ¬ | ¬ |
| Benn et al., 2016 | Lung cancer | ¬ | ¬ | ¬ | ¬ | ¬ | ¬ | – | ¬ | ¬ | ¬ |
| Zhou et al., 2021 | Lung cancer | ¬ | ¬ | ¬ | ¬ | ¬ | ¬ | ¬ | ¬ | ¬ | ¬ |
| Vithayathil et al., 2021 | Lung cancer | ¬ | ¬ | ¬ | ¬ | ¬ | ¬ | ¬ | ¬ | ¬ | ¬ |
| Dusingize et al., 2020 | Melanoma | ¬ | ¬ | ¬ | ¬ | ¬ | ¬ | ¬ | ¬ | ¬ | ¬ |
| Vithayathil et al., 2021 | Melanoma | ¬ | ¬ | ¬ | ¬ | ¬ | ¬ | ¬ | ¬ | ¬ | ¬ |
| Benn et al., 2016 | Non-melanoma | ¬ | ¬ | ¬ | ¬ | ¬ | ¬ | – | ¬ | ¬ | ¬ |
| Vithayathil et al., 2021 | Thyroid cancer | ¬ | ¬ | ¬ | ¬ | ¬ | ¬ | ¬ | ¬ | ¬ | ¬ |

*Study quality was assessed using a modified version of Strengthening the Reporting of Mendelian Randomisation Studies (STROBE-MR) guidelines [1, 2]. A star was given if the following was satisfied:

1. The title and/or abstract indicate Mendelian randomization design.

2. The background and rationale for the study and the objective are clearly reported.

3. The study design and data source(s) used are clearly reported.

4. The study sample, including the number of cases and non-cases or total number of participants included in the analysis, is reported.

5. The selection of genetic variants as well as the number of genetic variants used in the Mendelian randomization analysis are reported.

6. The statistical methods used for the primary analysis and the exposure unit are reported.

7. Sensitivity analyses based on robust Mendelian randomization methods (e.g., the weighted median and/or MR-Egger regression) were conducted and reported.

8. The software used for the Mendelian randomization analysis is reported.

9. Relative risk (odds ratio) estimates are clearly presented in tables or figures.

10. The limitations of the study are discussed and the overall interpretation of results considering the objective and limitations is sound.

**References**

1. Smith GD, Davies NM, Egger M, Gallo V, Golub R, Higgins JP, Langenberg C, Loder EW, Richards JB, C. RR et al: STROBE-MR: Guidelines for strengthening the reporting of Mendelian randomization studies, PeerJ Preprints Jul 15 2019; 7:e27857v1.

2. Burgess S, Davey Smith G, Davies NM, Dudbridge F, Gill D, Glymour MM, Hartwig FP, Holmes MV, Minelli C, Relton CL et al: Guidelines for performing Mendelian randomization investigations. Wellcome Open Res 2019, 4:186.

**Table S2.** Mendelian randomization studies included in the meta-analyses of genetically predicted body mass index in relation to diabetes mellitus, diseases of the circulatory, respiratory, digestive, musculoskeletal, and nervous systems, and neoplasms

| **Disease group** | **Reference** | **Study** | **Outcome** | **Cases** | **Non-cases** | **Total, N** | **SNPs** | **Unit** | **OR** | **LB** | **UB** | ***P*** | **I^2^** | **Population** |
| --- | --- | --- | --- | --- | --- | --- | --- | --- | --- | --- | --- | --- | --- | --- |
| Diabetes | Hyppönen et al., 2019 | UK Biobank | Type 1 diabetes mellitus | 2258 | 316133 | 318391 | 76 | SD | 2.11 | 1.62 | 2.76 |  |  | European |
| Diabetes | De novo analysis, 2021 | FinnGen | Type 1 diabetes mellitus | 2649 | 183674 | 186323 | 305 | SD | 1.05 | 0.85 | 1.31 |  |  | European |
| Diabetes |  | **Meta-analysis** | **Type 1 diabetes mellitus** | **4907** | **499807** | **504714** |  | SD | **1.39** | **1.17** | **1.64** | **<0.001** | **94%** | European |
| Diabetes | Yuan & Larsson SC, 2020 | DIAGRAM, UK Biobank | Type 2 diabetes mellitus | 74124 | 824006 | 898130 | 661 | SD | 1.89 | 1.73 | 2.07 |  |  | European |
| Diabetes | De novo analysis, 2021 | FinnGen | Type 2 diabetes mellitus | 29166 | 183185 | 212351 | 305 | SD | 2.50 | 2.14 | 2.91 |  |  | European |
| Diabetes |  | **Meta-analysis** | **Type 2 diabetes mellitus** | **103290** | **1007191** | **1110481** |  | SD | **2.03** | **1.88** | **2.19** | **<0.001** | **90%** | European |
| Circulatory | van 't Hof, et al., 2017 | Dutch studies | Aortic aneurysm | 818 | 3004 | 3822 | 97 | SD | 1.63 | 0.99 | 2.61 |  |  | European |
| Circulatory | Larsson et al., 2020 | UK Biobank | Aortic aneurysm | 758 | 366945 | 367703 | 96 | SD | 1.30 | 0.83 | 2.06 |  |  | European |
| Circulatory | De novo analysis, 2021 | FinnGen | Aortic aneurysm | 2825 | 206541 | 209366 | 305 | SD | 1.62 | 1.30 | 2.01 |  |  | European |
| Circulatory |  | **Meta-analysis** | **Aortic aneurysm** | **4401** | **576490** | **580891** |  | SD | **1.57** | **1.31** | **1.88** | **<0.001** | **0%** | European |
| Circulatory | Kaltoft et al., 2020 | CGPS | Aortic valve stenosis | 1215 | 106996 | 108211 | 5 | 1 kg/m^2^ | 1.45* | 1.19 | 1.78 |  |  | European |
| Circulatory | Larsson et al., 2020 | UK Biobank | Aortic valve stenosis | 1252 | 366451 | 367703 | 96 | SD | 1.76 | 1.25 | 2.48 |  |  | European |
| Circulatory |  | **Meta-analysis** | **Aortic valve stenosis** | **2467** | **473447** | **475914** |  | SD | **2.02** | **1.46** | **2.79** | **<0.001** | **82%** | European |
| Circulatory | Chatterjee, et al., 2017 | Seven cohorts | Atrial fibrillation | 4178 | 51646 | 55824 | 2 | 1 kg/m^2^ | 1.11 | 1.05 | 1.17 |  |  | European |
| Circulatory | Larsson et al., 2020 | UK Biobank | Atrial fibrillation | 13538 | 354165 | 367703 | 96 | SD | 1.35 | 1.20 | 1.52 |  |  | European |
| Circulatory | De novo analysis, 2021 | FinnGen | Atrial fibrillation | 22068 | 116926 | 138994 | 305 | SD | 1.98 | 1.75 | 2.24 |  |  | European |
| Circulatory |  | **Meta-analysis** | **Atrial fibrillation** | **39784** | **522737** | **562521** |  | SD | **1.62** | **1.50** | **1.76** | **<0.001** | **90%** | European |
| Circulatory | Gill et al., 2021 | CARDIoGRAMplusC4D | Coronary artery disease | 60801 | 123504 | 184305 | NA | SD | 1.49 | 1.39 | 1.60 |  |  | Trans-ancestry |
| Circulatory | Nordestgaard et al., 2012 | CGPS, CCHS | Coronary artery disease | 11056 | 64571 | 75627 | 3 | SD | 1.52 | 1.12 | 2.05 |  |  | European |
| Circulatory | Larsson et al., 2020 | UK Biobank | Coronary artery disease | 24531 | 343172 | 367703 | 96 | SD | 1.35 | 1.18 | 1.55 |  |  | European |
| Circulatory | De novo analysis, 2021 | FinnGen | Coronary artery disease | 30952 | 187840 | 218792 | 305 | SD | 1.39 | 1.27 | 1.52 |  |  | European |
| Circulatory |  | **Meta-analysis** | **Coronary artery disease** | **127340** | **719087** | **846427** |  | SD | **1.44** | **1.37** | **1.51** | **<0.001** | **0%** | Trans-ancestry |
| Circulatory | Klovaite et al., 2015 | CGPS, CCHS | Deep vein thrombosis | 1815 | 85759 | 87574 | 1 | 1 kg/m^2^ | 1.13 | 0.92 | 1.39 |  |  | European |
| Circulatory | Larsson et al., 2020 | UK Biobank | Deep vein thrombosis | 8891 | 358812 | 367703 | 96 | SD | 1.56 | 1.37 | 1.79 |  |  | European |
| Circulatory | De novo analysis, 2021 | FinnGen | Deep vein thrombosis | 7988 | 210804 | 218792 | 305 | SD | 1.64 | 1.38 | 1.94 |  |  | European |
| Circulatory |  | **Meta-analysis** | **Deep vein thrombosis** | **18694** | **655375** | **674069** |  | SD | **1.59** | **1.43** | **1.77** | **<0.001** | **0%** | European |
| Circulatory | Shah et al., 2020 | HERMES, UK Biobank | Heart failure | 47309 | 930014 | 977323 | 89 | SD | 1.61 | 1.45 | 1.79 |  |  | European |
| Circulatory | De novo analysis, 2021 | FinnGen | Heart failure | 13087 | 195091 | 208178 | 305 | SD | 1.78 | 1.60 | 1.99 |  |  | European |
| Circulatory |  | **Meta-analysis** | **Heart failure** | **60396** | **1125105** | **1185501** |  | SD | **1.69** | **1.57** | **1.82** | **<0.001** | **41%** | European |
| Circulatory | Giontella et al., 2021 | MDC study | Hypertension | NA | NA | 29262 | 565 | SD | 1.56 | 1.39 | 1.75 |  |  | European |
| Circulatory | Giontella et al., 2021 | MPP | Hypertension | NA | NA | 9137 | 565 | SD | 1.48 | 1.19 | 1.83 |  |  | European |
| Circulatory | Larsson et al., 2020 | UK Biobank | Hypertension | 119500 | 248203 | 367703 | 96 | SD | 1.55 | 1.40 | 1.73 |  |  | European |
| Circulatory | De novo analysis, 2021 | FinnGen | Hypertension | 42857 | 162837 | 205694 | 305 | SD | 1.91 | 1.75 | 2.09 |  |  | European |
| Circulatory |  | **Meta-analysis** | **Hypertension** | **162357** | **411040** | **611796** |  | SD | **1.68** | **1.59** | **1.78** | **<0.001** | **77%** | European |
| Circulatory | Harshfield et al., 2021 | ISGC | Intracerebral hemorrhage | 1545 | 1481 | 3026 | 505 | SD | 1.26 | 0.90 | 1.75 |  |  | European |
| Circulatory | Larsson et al., 2020 | UK Biobank | Intracerebral hemorrhage | 1655 | 366048 | 367703 | 96 | SD | 1.15 | 0.85 | 1.56 |  |  | European |
| Circulatory | De novo analysis, 2021 | FinnGen | Intracerebral hemorrhage | 1687 | 201146 | 202833 | 305 | SD | 1.23 | 0.94 | 1.60 |  |  | European |
| Circulatory |  | **Meta-analysis** | **Intracerebral hemorrhage** | **4887** | **568675** | **573562** |  | SD | **1.21** | **1.02** | **1.44** | **0.028** | **0%** | European |
| Circulatory | Harshfield et al., 2021 | MEGASTROKE | Ischemic stroke | 60341 | 454450 | 514791 | 505 | SD | 1.14 | 1.06 | 1.22 |  |  | European |
| Circulatory | Larsson et al., 2020 | UK Biobank | Ischemic stroke | 3554 | 364149 | 367703 | 96 | SD | 1.14 | 0.93 | 1.40 |  |  | European |
| Circulatory | De novo analysis, 2021 | FinnGen | Ischemic stroke | 10551 | 202223 | 212774 | 305 | SD | 1.23 | 1.09 | 1.38 |  |  | European |
| Circulatory |  | **Meta-analysis** | **Ischemic stroke** | **74446** | **1020822** | **1095268** |  | SD | **1.16** | **1.10** | **1.23** | **<0.001** | **0%** | European |
| Circulatory | Huang et al., 2016 | Shanghai cohort | Peripheral artery disease | 701 | 10776 | 11477 | 14 | 1 kg/m^2^† | 1.44 | 1.18 | 1.75 |  |  | Chinese |
| Circulatory | Gill et al., 2021 | MVP | Peripheral artery disease | 31307 | 211753 | 243060 | NA | SD | 1.70 | 1.58 | 1.82 |  |  | European |
| Circulatory | Larsson et al., 2020 | UK Biobank | Peripheral artery disease | 3514 | 364189 | 367703 | 96 | SD | 1.39 | 1.14 | 1.71 |  |  | European |
| Circulatory | De novo analysis, 2021 | FinnGen | Peripheral artery disease | 7098 | 206541 | 213639 | 305 | SD | 1.57 | 1.36 | 1.81 |  |  | European |
| Circulatory |  | **Meta-analysis** | **Peripheral artery disease** | **42620** | **793259** | **835879** |  | SD | **1.66** | **1.56** | **1.76** | **<0.001** | **58%** | Trans-ancestry |
| Circulatory |  | **Meta-analysis** | **Peripheral artery disease** | **41919** | **782483** | **824402** |  | SD | **1.65** | **1.55** | **1.75** | **<0.001** | **49%** | European |
| Circulatory | Larsson et al., 2020 | UK Biobank | Pulmonary embolism | 5097 | 362606 | 367703 | 96 | SD | 1.34 | 1.11 | 1.62 |  |  | European |
| Circulatory | De novo analysis, 2021 | FinnGen | Pulmonary embolism | 4185 | 214228 | 218413 | 305 | SD | 1.34 | 1.13 | 1.59 |  |  | European |
| Circulatory |  | **Meta-analysis** | **Pulmonary embolism** | **9282** | **576834** | **586116** |  | SD | **1.34** | **1.18** | **1.52** | **<0.001** | **0%** | European |
| Circulatory | Karhunen et al., 2021 | ISGC | Subarachnoid hemorrhage | 4196 | 59544 | 63740 | 967 | SD | 1.21 | 1.03 | 1.41 |  |  | European |
| Circulatory | Larsson et al., 2020 | UK Biobank | Subarachnoid hemorrhage | 1834 | 365869 | 367703 | 96 | SD | 1.33 | 1.00 | 1.77 |  |  | European |
| Circulatory | De novo analysis, 2021 | FinnGen | Subarachnoid hemorrhage | 1338 | 201230 | 202568 | 305 | SD | 1.05 | 0.79 | 1.40 |  |  | European |
| Circulatory |  | **Meta-analysis** | **Subarachnoid hemorrhage** | **7368** | **626643** | **634011** |  | SD | **1.20** | **1.06** | **1.36** | **0.004** | **0%** | European |
| Circulatory | Larsson et al., 2020 | UK Biobank | Transient ischemic attack | 3485 | 364218 | 367703 | 96 | SD | 1.08 | 0.86 | 1.35 |  |  | European |
| Circulatory | De novo analysis, 2021 | FinnGen | Transient ischemic attack | 8835 | 202223 | 211058 | 305 | SD | 1.21 | 1.07 | 1.37 |  |  | European |
| Circulatory |  | **Meta-analysis** | **Transient ischemic attack** | **12320** | **566441** | **578761** |  | SD | **1.18** | **1.06** | **1.31** | **0.003** | **0%** | European |
| Circulatory | Lindström et al., 2017 | INVENT | Venous thromboembolism | 7507 | 52632 | 60139 | 95 | SD | 1.59 | 1.30 | 1.93 |  |  | European |
| Circulatory | De novo analysis, 2021 | FinnGen | Venous thromboembolism | 9176 | 209616 | 218792 | 305 | SD | 1.44 | 1.26 | 1.64 |  |  | European |
| Circulatory |  | **Meta-analysis** | **Venous thromboembolism** | **16683** | **262248** | **278931** |  | SD | **1.49** | **1.33** | **1.66** | **<0.001** | **0%** | European |
| Respiratory | Sun et al., 2020 | HUNT study | Asthma | 4987 | 51118 | 56105 | 73 | SD | 1.36 | 1.10 | 1.68 |  |  | European |
| Respiratory | Au Yeung et al., 2021 | UK Biobank | Asthma | 26332 | 375505 | 401837 | 323 | SD | 1.33 | 1.25 | 1.43 |  |  | European |
| Respiratory | Au Yeung et al., 2021 | GABRIEL consortium | Asthma | 1947 | 3669 | 5616 | 115 | SD | 1.17 | 0.77 | 1.77 |  |  | European |
| Respiratory | De novo analysis, 2021 | FinnGen | Asthma | 20629 | 135449 | 156078 | 305 | SD | 1.44 | 1.30 | 1.59 |  |  | European |
| Respiratory |  | **Meta-analysis** | **Asthma** | **53895** | **565741** | **619636** |  | **SD** | **1.36** | **1.29** | **1.43** | **<0.001** | **0%** | European |
| Respiratory | Hyppönen et al, 2019 | UK Biobank | COPD | 6607 | 304193 | 310800 | 76 | SD | 1.52 | 1.23 | 1.87 |  |  | European |
| Respiratory | De novo analysis, 2021 | FinnGen | COPD | 6916 | 186723 | 193639 | 305 | SD | 1.71 | 1.48 | 1.96 |  |  | European |
| Respiratory |  | **Meta-analysis** | **COPD** | **13523** | **490916** | **504439** |  | **SD** | **1.65** | **1.47** | **1.85** | **<0.001** | **0%** | European |
| Digestive | Yuan & Larsson., 2021 | UK Biobank | Diverticular disease | 12662 | 348532 | 361194 | 312 | SD | 1.26 | 1.14 | 1.41 |  |  | European |
| Digestive | Yuan & Larsson., 2021 | FinnGen | Diverticular disease | 10978 | 149001 | 159979 | 312 | SD | 1.19 | 1.06 | 1.34 |  |  | European |
| Digestive |  | **Meta-analysis** | **Diverticular disease** | **23640** | **497533** | **521173** |  | **SD** | **1.23** | **1.13** | **1.33** | **<0.001** | **0%** | European |
| Digestive | Stender et al., 2013 | CPGS | Gallstone disease | 4106 | 73573 | 77679 | 3 | 1 kg/m^2^ | 1.17 | 0.99 | 1.37 |  |  | European |
| Digestive | Pang et al., 2020 | China Kadoorie Biobank | Gallstone disease | NA | NA | 473938 | 92 | SD | 1.62 | 1.17 | 2.24 |  |  | Chinese |
| Digestive | Yuan et al., 2021 | UK Biobank | Gallstone disease | 10520 | 350674 | 361194 | 97 | SD | 1.61 | 1.43 | 1.81 |  |  | European |
| Digestive | Yuan et al., 2021 | FinnGen | Gallstone disease | 11675 | 121348 | 133023 | 97 | SD | 1.67 | 1.43 | 1.94 |  |  | European |
| Digestive |  | **Meta-analysis** | **Gallstone disease** | **26301** | **545595** | **1045834** |  | **SD** | **1.64** | **1.50** | **1.79** | **<0.001** | **0%** | Trans-ancestry |
| Digestive |  | **Meta-analysis** | **Gallstone disease** | **26301** | **1017617** | **1043918** |  | **SD** | **1.64** | **1.49** | **1.80** | **<0.001** | **0%** | European |
| Digestive | Green et al., 2020 | UK Bioank | GERD | NA | NA | 378214 | 72 | SD | 1.06 | 0.96 | 1.17 |  |  | European |
| Digestive | De novo analysis, 2021 | FinnGen | GERD | 13141 | 189695 | 202836 | 305 | SD | 1.20 | 1.08 | 1.34 |  |  | European |
| Digestive |  | **Meta-analysis** | **GERD** | **NA** | **NA** | **581050** |  | **SD** | **1.12** | **1.04** | **1.21** | **0.002** | **64%** | European |
| Digestive | Carreras-Torres et al., 2020 | IBD consortium | IBD-Crohn's disease | 12194 | 34915 | 47109 | 816 | SD | 1.11 | 1.02 | 1.20 |  |  | European |
| Digestive | De novo analysis, 2021 | FinnGen | IBD-Crohn's disease | 657 | 210300 | 210957 | 305 | SD | 1.28 | 0.85 | 1.91 |  |  | European |
| Digestive |  | **Meta-analysis** | **IBD-Crohn's disease** | **12851** | **245215** | **258066** |  | **SD** | **1.12** | **1.03** | **1.21** | **0.007** | **0%** | European |
| Digestive | Carreras-Torres et al., 2020 | IBD consortium | IBD-Ulcerative colitis | 12366 | 34915 | 47281 | 816 | SD | 0.85 | 0.78 | 0.92 |  |  | European |
| Digestive | De novo analysis, 2021 | FinnGen | IBD-Ulcerative colitis | 2251 | 210300 | 212551 | 305 | SD | 0.90 | 0.71 | 1.14 |  |  | European |
| Digestive |  | **Meta-analysis** | **IBD-Ulcerative colitis** | **14617** | **245215** | **259832** |  | **SD** | **0.86** | **0.79** | **0.92** | **<0.001** | **0%** | European |
| Digestive | Pang et al., 2020 | China Kadoorie Biobank | NAFLD | NA | NA | 473938 | 92 | SD | 1.79 | 0.61 | 5.24 |  |  | Chinese |
| Digestive | De novo analysis, 2021 | FinnGen | NAFLD | 894 | 217898 | 218792 | 305 | SD | 1.81 | 1.25 | 2.93 |  |  | European |
| Digestive |  | **Meta-analysis** | **NAFLD** | **894** | **217898** | **692730** |  | **SD** | **1.81** | **1.22** | **2.69** | **0.003** | **0%** | Trans-ancestry |
| Musculoskeletal | Majeed et al., 2021 | Dupuytren’s disease GWAS | Dupuytren’s disease | NA | NA | NA | 774 | SD | 0.72 | 0.63 | 0.82 |  |  | European |
| Musculoskeletal | De novo analysis, 2021 | FinnGen | Dupuytren’s disease | 2588 | 167641 | 170229 | 305 | SD | 0.97 | 0.77 | 1.23 |  |  | European |
| Musculoskeletal |  | **Meta-analysis** | **Dupuytren’s disease** | **NA** | **NA** | **NA** |  | **SD** | **0.77** | **0.69** | **0.87** | **<0.001** | **79%** | **European** |
| Musculoskeletal | Larsson et al., 2018 | GUGC | Gout | 2115 | 67259 | 69374 | 97 | SD | 2.24 | 1.70 | 2.95 |  |  | European |
| Musculoskeletal | De novo analysis, 2021 | FinnGen | Gout | 3576 | 203546 | 207122 | 305 | SD | 1.71 | 1.35 | 2.17 |  |  | European |
| Musculoskeletal |  | **Meta-analysis** | **Gout** | **5691** | **270805** | **276496** |  | **SD** | **1.92** | **1.60** | **2.30** | **<0.001** | **53%** | European |
| Musculoskeletal | Hindy et al., 2019 | MDCS | Osteoarthritis‡ | 3852 | 21487 | 25339 | 31 | SD | 1.24 | 0.86 | 1.79 |  |  | European |
| Musculoskeletal | Funck-Brentano et al., 2019 | UK Biobank | Osteoarthritis | 48431 | 336407 | 384838 | NA | SD | 1.57 | 1.44 | 1.71 |  |  | European |
| Musculoskeletal |  | **Meta-analysis** | **Osteoarthritis** | **51990** | **360539** | **412529** |  | **SD** | **1.55** | **1.43** | **1.69** | **<0.001** | **34%** | European |
| Musculoskeletal | Tang et al., 2021 | RA GWAS | Rheumatoid arthritis | 14361 | 43923 | 58284 | 670 | SD | 1.22 | 1.09 | 1.37 |  |  | European |
| Musculoskeletal | De novo analysis, 2021 | FinnGen | Rheumatoid arthritis | 6236 | 147221 | 153457 | 305 | SD | 1.36 | 1.18 | 1.56 |  |  | European |
| Musculoskeletal |  | **Meta-analysis** | **Rheumatoid arthritis** | **20597** | **191144** | **211741** |  | **SD** | **1.27** | **1.17** | **1.39** | **<0.001** | **28%** | European |
| Musculoskeletal | De novo analysis, 2021 | FinnGen | Osteoporosis | 3203 | 209575 | 212778 | 305 | SD | 0.81 | 0.65 | 0.99 | 0.044 |  | European |
| Nervous | Larsson, et al. 2017 | IGAP | Alzheimer's disease | 17008 | 37154 | 54162 | 76 | SD | 1.05 | 0.91 | 1.21 |  |  | European |
| Nervous | Nordestgaard et al., 2017 | CPGS | Alzheimer's disease | 645 | 94933 | 95578 | 5 | 1 kg/m^2^ | 1.02§ | 0.81 | 1.30 |  |  | European |
| Nervous | De novo analysis, 2021 | FinnGen | Alzheimer's disease | 3899 | 214893 | 218792 | 305 | SD | 1.16 | 0.97 | 1.39 |  |  | European |
| Nervous |  | **Meta-analysis** | **Alzheimer's disease** | **21552** | **346980** | **368532** |  | **SD** | **1.09** | **0.98** | **1.22** | **0.124** | **0%** | European |
| Nervous | Zeng et al., 2019 | AVS GWAS | ALS | 20806 | 59804 | 80610 | 1031 | SD | 1.04 | 0.97 | 1.11 |  |  | European |
| Nervous | De novo analysis, 2021 | FinnGen | ALS | 219 | 111621 | 111840 | 305 | SD | 1.22 | 0.62 | 2.39 |  |  | European |
| Nervous |  | **Meta-analysis** | **ALS** | **21025** | **171425** | **192450** |  | **SD** | **1.04** | **0.97** | **1.11** | **0.233** | **0%** | European |
| Nervous | Yuan et al., 2021 | IMSGC | Multiple sclerosis | 14498 | 24091 | 38589 | 963 | SD | 1.27 | 1.15 | 1.41 |  |  | European |
| Nervous | De novo analysis, 2021 | FinnGen | Multiple sclerosis | 1048 | 217141 | 218189 | 305 | SD | 1.14 | 0.82 | 1.59 |  |  | European |
| Nervous |  | **Meta-analysis** | **Multiple sclerosis** | **15546** | **241232** | **256778** |  | **SD** | **1.26** | **1.14** | **1.39** | **<0.001** | **0%** | European |
| Nervous | Noyce et al., 2019 | PD GWAS | Parkinson's disease | 26035 | 403190 | 429225 | 300 | SD | 0.96 | 0.83 | 1.12 |  |  | European |
| Nervous | De novo analysis, 2021 | FinnGen | Parkinson's disease | 2162 | 216630 | 218792 | 305 | SD | 0.76 | 0.60 | 0.96 |  |  | European |
| Nervous |  | **Meta-analysis** | **Parkinson's disease** | **28197** | **619820** | **648017** |  | **SD** | **0.90** | **0.79** | **1.02** | **0.093** | **63%** | European |
| Neoplasm | Bull et al., 2020 | GECCO, CCTS, CCFR | Colorectal cancer | 58221 | 67984 | 126205 | 312 | SD | 1.16 | 1.07 | 1.26 |  |  | European |
| Neoplasm | Benn et al., 2016 | CGPS, CCHS | Colorectal cancer | 1499 | 95959 | 97458 | 5 | 10 | 1.18 | 0.23 | 6.20 |  |  | European |
| Neoplasm | Suzuki et al., 2021 | Japanese cohorts | Colorectal cancer | 7636 | 37141 | 44777 | 654 | SD | 1.07 | 1.03 | 1.11 |  |  | Japanese |
| Neoplasm | Vithayathil et al., 2021 | UK Biobank | Colorectal cancer | 6995 | 360566 | 367561 | 312 | SD | 1.06 | 0.93 | 1.20 |  |  | European |
| Neoplasm | De novo analysis, 2021 | FinnGen | Colorectal cancer | 3022 | 215770 | 218792 | 305 | SD | 1.12 | 0.91 | 1.37 |  |  | European |
| Neoplasm |  | **Meta-analysis** | **Colorectal cancer** | **77373** | **777420** | **854793** |  | **SD** | **1.08** | **1.05** | **1.12** | **<0.001** | **0%** | Trans-ancestry |
| Neoplasm |  | **Meta-analysis** | **Colorectal cancer** | **69737** | **740279** | **810016** |  | **SD** | **1.13** | **1.06** | **1.20** | **<0.001** | **0%** | European |
| Neoplasm | Thrift et al., 2012 | BEAGSS | Esophageal cancer | 999 | 2169 | 3168 | 29 | 1 kg/m^2^ | 1.23 | 1.06 | 1.43 |  |  | European |
| Neoplasm | Vithayathil et al., 2021 | UK Biobank | Esophageal cancer | 1228 | 366333 | 367561 | 312 | SD | 1.56 | 1.16 | 2.11 |  |  | European |
| Neoplasm | De novo analysis, 2021 | FinnGen | Esophageal cancer | 232 | 218560 | 218792 | 305 | SD | 2.17 | 1.08 | 4.37 |  |  | European |
| Neoplasm |  | **Meta-analysis** | **Esophageal cancer** | **2459** | **587062** | **589521** |  | **SD** | **1.75** | **1.35** | **2.62** | **<0.001** | **14%** | European |
| Neoplasm | Barahona Ponce et al., 2020 | Chilean study | Gallbladder cancer | 277 | 2107 | 2384 | 289 | SD | 2.47 | 1.10 | 5.54 |  |  | Chilean |
| Neoplasm | Barahona Ponce et al., 2020 | European cohorts | Gallbladder cancer | 103 | 168 | 271 | 289 | SD | 0.91 | 0.22 | 3.78 |  |  | European |
| Neoplasm | Vithayathil et al., 2021 | UK Biobank | Gallbladder cancer | 604 | 366957 | 367561 | 312 | SD | 1.39 | 0.93 | 2.08 |  |  | European |
| Neoplasm |  | **Meta-analysis** | **Gallbladder cancer** | **984** | **369232** | **370216** |  | **SD** | **1.50** | **1.06** | **2.14** | **0.021** | **4%** | European |
| Neoplasm | Pang et al., 2020 | China Kadoorie Biobank | Liver cancer | NA | NA | NA | 92 | SD | 1.70 | 0.93 | 3.10 |  |  | Chinese |
| Neoplasm | Vithayathil et al., 2021 | UK Biobank | Liver cancer | 463 | 367098 | 367561 | 312 | SD | 1.81 | 1.14 | 2.88 |  |  | European |
| Neoplasm | De novo analysis, 2021 | FinnGen | Liver cancer | 304 | 218488 | 218792 | 305 | SD | 1.31 | 0.73 | 2.35 |  |  | European |
| Neoplasm |  | **Meta-analysis** | **Liver cancer** | **NA** | **NA** | **NA** |  | **SD** | **1.62** | **1.19** | **2.22** | **0.002** | **0%** | Trans-ancestry |
| Neoplasm |  | **Meta-analysis** | **Liver cancer** | **767** | **804074** | **1172706** |  | **SD** | **1.60** | **1.11** | **2.30** | **0.011** | **0%** | European |
| Neoplasm | Lu et al., 2020 | PanScan, PanC4 | Pancreatic cancer | 8769 | 7066 | 15835 | 276 | SD | 1.38 | 1.17 | 1.64 |  |  | European |
| Neoplasm | Vithayathil et al., 2021 | UK Biobank | Pancreatic cancer | 1747 | 365814 | 367561 | 312 | SD | 1.34 | 1.06 | 1.70 |  |  | European |
| Neoplasm | De novo analysis, 2021 | FinnGen | Pancreatic cancer | 605 | 218187 | 218792 | 305 | SD | 1.32 | 0.87 | 2.00 |  |  | European |
| Neoplasm |  | **Meta-analysis** | **Pancreatic cancer** | **11121** | **591067** | **602188** |  | **SD** | **1.36** | **1.20** | **1.55** | **<0.001** | **0%** | European |
| Neoplasm | Mao et al., 2017 | Nanjing/Beijing | Stomach cancer | 2631 | 4373 | 7004 | 37 | SD | 1.07 | 1.02 | 1.13 |  |  | Chinese |
| Neoplasm | Vithayathil et al., 2021 | UK Biobank | Stomach cancer | 994 | 366567 | 367561 | 312 | SD | 1.80 | 1.31 | 2.47 |  |  | European |
| Neoplasm | De novo analysis, 2021 | FinnGen | Stomach cancer | 633 | 218159 | 218792 | 305 | SD | 1.41 | 0.94 | 2.12 |  |  | European |
| Neoplasm |  | **Meta-analysis** | **Stomach cancer** | **4258** | **589099** | **593357** |  | **SD** | **1.09** | **1.04** | **1.14** | **0.001** | **83%** | Trans-ancestry |
| Neoplasm |  | **Meta-analysis** | **Stomach cancer** | **1627** | **584726** | **586353** |  | **SD** | **1.64** | **1.28** | **2.11** | **<0.001** | **0%** | European |
| Neoplasm | Benn et al., 2016 | CGPS, CCHS | Breast cancer | 507 | 19975 | 20482 | 5 | 10 | 0.47 | 0.14 | 1.51 |  |  | European |
| Neoplasm | Benn et al., 2016 | CGPS, CCHS | Breast cancer | 2351 | 30665 | 33016 | 5 | 10 | 1.11 | 0.34 | 3.59 |  |  | European |
| Neoplasm | Vithayathil et al., 2021 | BCAC | Breast cancer | 122977 | 105974 | 228951 | 297 | SD | 0.84 | 0.77 | 0.91 |  |  | European |
| Neoplasm | Vithayathil et al., 2021 | UK Biobank | Breast cancer | 15695 | 183130 | 198825 | 312 | SD | 0.96 | 0.86 | 1.06 |  |  | European |
| Neoplasm | De novo analysis, 2021 | FinnGen | Breast cancer | 8401 | 115178 | 123579 | 305 | SD | 0.77 | 0.68 | 0.88 |  |  | European |
| Neoplasm |  | **Meta-analysis** | **Breast cancer** | **149931** | **454922** | **604853** |  | **SD** | **0.87** | **0.82** | **0.92** | **<0.001** | **57%** | European |
| Neoplasm | Mazuda et al. 2020 | BioBank Japan | Cervical cancer | 538 | 39556 | 40094 | 74 | SD | 1.02 | 0.87 | 1.19 |  |  | Japanese |
| Neoplasm | Vithayathil et al., 2021 | UK Biobank | Cervical cancer | 1973 | 196852 | 198825 | 312 | SD | 1.21 | 0.96 | 1.53 |  |  | European |
| Neoplasm | De novo analysis, 2021 | FinnGen | Cervical cancer | 1648 | 121931 | 123579 | 305 | SD | 1.37 | 1.06 | 1.77 |  |  | European |
| Neoplasm |  | **Meta-analysis** | **Cervical cancer** | **3621** | **318783** | **322404** |  | **SD** | **1.13** | **1.01** | **1.27** | **0.039** | **52%** | Trans-ancestry |
| Neoplasm |  | **Meta-analysis** | **Cervical cancer** | **3083** | **279227** | **282310** |  | **SD** | **1.28** | **1.08** | **1.52** | **0.005** | **56%** | European |
| Neoplasm | Mazuda et al. 2020 | BioBank Japan | Endometrial cancer | 909 | 39556 | 40465 | 74 | SD | 1.22 | 1.08 | 1.38 |  |  | Japanese |
| Neoplasm | Vithayathil et al., 2021 | ECAC, UK Biobank | Endometrial cancer | 12906 | 108979 | 121885 | 312 | SD | 1.81 | 1.60 | 2.03 |  |  | European |
| Neoplasm | De novo analysis, 2021 | FinnGen | Endometrial cancer | 1053 | 122526 | 123579 | 305 | SD | 1.48 | 1.06 | 2.08 |  |  | European |
| Neoplasm |  | **Meta-analysis** | **Endometrial cancer** | **13959** | **231505** | **245464** |  | **SD** | **1.49** | **1.38** | **1.62** | **<0.001** | **90%** | Trans-ancestry |
| Neoplasm |  | **Meta-analysis** | **Endometrial cancer** | **13050** | **191949** | **369043** |  | **SD** | **1.77** | **1.58** | **1.90** | **<0.001** | **18%** | European |
| Neoplasm | Mazuda et al. 2020 | BioBank Japan | Ovarian cancer | 647 | 39556 | 40203 | 74 | SD | 1.12 | 0.97 | 1.29 |  |  | Japanese |
| Neoplasm | Vithayathil et al., 2021 | OCAC | Ovarian cancer | 25509 | 40941 | 66450 | 297 | SD | 1.07 | 0.95 | 1.19 |  |  | European |
| Neoplasm | Vithayathil et al., 2021 | UK Biobank | Ovarian cancer | 1839 | 196986 | 198825 | 312 | SD | 1.22 | 0.95 | 1.56 |  |  | European |
| Neoplasm | De novo analysis, 2021 | FinnGen | Ovarian cancer | 719 | 122860 | 123579 | 305 | SD | 0.76 | 0.52 | 1.12 |  |  | European |
| Neoplasm |  | **Meta-analysis** | **Ovarian cancer** | **28067** | **360787** | **388854** |  | **SD** | **1.09** | **1.00** | **1.18** | **0.050** | **32%** | Trans-ancestry |
| Neoplasm |  | **Meta-analysis** | **Ovarian cancer** | **27420** | **321231** | **348651** |  | **SD** | **1.07** | **0.97** | **1.18** | **0.193** | **52%** | European |
| Neoplasm | Vithayathil et al., 2021 | UK Biobank | Bladder cancer | 3326 | 364235 | 367561 | 312 | SD | 1.15 | 0.95 | 1.38 |  |  | European |
| Neoplasm | De novo analysis, 2021 | FinnGen | Bladder cancer | 1115 | 217677 | 218792 | 305 | SD | 1.72 | 1.25 | 2.38 |  |  | European |
| Neoplasm |  | **Meta-analysis** | **Bladder cancer** | **4441** | **581912** | **586353** |  | **SD** | **1.27** | **1.03** | **1.50** | **0.003** | **78%** | European |
| Neoplasm | Benn et al., 2016 | CGPS, CCHS | Kidney cancer | 193 | 97265 | 97458 | 5 | 10 kg/m^2^ | 1.00 | 0.14 | 6.73 |  |  | European |
| Neoplasm | Johansson et al., 2019 | GWAS meta-analysis | Kidney cancer | 10784 | 20406 | 31190 | 709 | SD | 1.56 | 1.44 | 1.70 |  |  | European |
| Neoplasm | Vithayathil et al., 2021 | UK Biobank | Kidney cancer | 1741 | 365820 | 367561 | 312 | SD | 1.27 | 1.00 | 1.63 |  |  | European |
| Neoplasm | De novo analysis, 2021 | FinnGen | Kidney cancer | 971 | 217821 | 218792 | 305 | SD | 1.19 | 0.85 | 1.67 |  |  | European |
| Neoplasm |  | **Meta-analysis** | **Kidney cancer** | **13689** | **701312** | **715001** |  | **SD** | **1.49** | **1.38** | **1.60** | **<0.001** | **64%** | European |
| Neoplasm | Benn et al., 2016 | CGPS, CCHS | Prostate cancer | 1576 | 42382 | 43958 | 5 | 10 kg/m^2^ | 2.46 | 0.34 | 18 |  |  | European |
| Neoplasm | Vithayathil et al., 2021 | PRACTICAL | Prostate cancer | 79148 | 61106 | 140254 | 300 | SD | 0.90 | 0.83 | 0.98 |  |  | European |
| Neoplasm | Vithayathil et al., 2021 | UK Biobank | Prostate cancer | 10506 | 158230 | 168736 | 312 | SD | 0.85 | 0.75 | 0.96 |  |  | European |
| Neoplasm | De novo analysis, 2021 | FinnGen | Prostate cancer | 6311 | 88902 | 95213 | 305 | SD | 0.94 | 0.80 | 1.10 |  |  | European |
| Neoplasm |  | **Meta-analysis** | **Prostate cancer** | **97541** | **350620** | **448161** |  | **SD** | **0.90** | **0.84** | **0.96** | **0.001** | **1%** | European |
| Neoplasm | Vithayathil et al., 2021 | UK Biobank | Testicular cancer | 747 | 167989 | 168736 | 312 | SD | 0.91 | 0.63 | 1.33 |  |  | European |
| Neoplasm | De novo analysis, 2021 | FinnGen | Testicular cancer | 199 | 95014 | 95213 | 305 | SD | 1.18 | 0.55 | 2.55 |  |  | European |
| Neoplasm |  | **Meta-analysis** | **Testicular cancer** | **946** | **263003** | **263949** |  | **SD** | **0.96** | **0.68** | **1.34** | **0.795** | **0%** | European |
| Neoplasm | Vithayathil et al., 2021 | UK Biobank | Leukemia, all | 1825 | 365736 | 367561 | 312 | SD | 1.15 | 0.91 | 1.45 |  |  | European |
| Neoplasm | De novo analysis, 2021 | FinnGen | Leukemia, lymphoid | 663 | 218129 | 218792 | 305 | SD | 0.90 | 0.61 | 1.35 |  |  | European |
| Neoplasm | De novo analysis, 2021 | FinnGen | Leukemia, myeloid | 283 | 218509 | 218792 | 305 | SD | 1.94 | 1.06 | 3.56 |  |  | European |
| Neoplasm |  | **Meta-analysis** | **Leukemia** | **2771** | **802374** | **805145** |  | **SD** | **1.14** | **0.95** | **1.39** | **0.115** | **54%** | European |
| Neoplasm | Went et al., 2020 | GWAS meta-analysis | Multiple myeloma | 7717 | 29304 | 37021 | 964 | SD | 1.10 | 0.99 | 1.22 |  |  | European |
| Neoplasm | Vithayathil et al., 2021 | UK Biobank | Multiple myeloma | 930 | 366631 | 367561 | 312 | SD | 1.00 | 0.72 | 1.39 |  |  | European |
| Neoplasm | De novo analysis, 2021 | FinnGen | Multiple myeloma | 598 | 218194 | 218792 | 305 | SD | 1.20 | 0.78 | 1.85 |  |  | European |
| Neoplasm |  | **Meta-analysis** | **Multiple myeloma** | **9245** | **614129** | **623374** |  | **SD** | **1.10** | **0.99** | **1.21** | **0.065** | **0%** | European |
| Neoplasm | Vithayathil et al., 2021 | UK Biobank | NHL, all | 2878 | 364683 | 367561 | 312 | SD | 1.19 | 0.99 | 1.44 |  |  | European |
| Neoplasm | De novo analysis, 2021 | FinnGen | NHL, follicular | 522 | 218270 | 218792 | 305 | SD | 1.06 | 0.68 | 1.66 |  |  | European |
| Neoplasm | De novo analysis, 2021 | FinnGen | NHL, non-follicular | 1283 | 180756 | 182039 | 305 | SD | 1.09 | 0.81 | 1.47 |  |  | European |
| Neoplasm |  | **Meta-analysis** | **NHL** | **4683** | **763709** | **768392** |  | **SD** | **1.15** | **0.99** | **1.33** | **0.069** | **0%** | European |
| Neoplasm | Saunders et al., 2020 | GWAS meta-analysis | Brain cancer | 12488 | 18169 | 30657 | 964 | SD | 0.96 | 0.88 | 1.06 |  |  | European |
| Neoplasm | Vithayathil et al., 2021 | UK Biobank | Brain cancer | 1057 | 366504 | 367561 | 312 | SD | 1.08 | 0.78 | 1.50 |  |  | European |
| Neoplasm | De novo analysis, 2021 | FinnGen | Brain cancer | 464 | 218328 | 218792 | 305 | SD | 0.74 | 0.45 | 1.20 |  |  | European |
| Neoplasm |  | **Meta-analysis** | **Brain cancer** | **14009** | **603001** | **617010** |  | **SD** | **0.96** | **0.88** | **1.05** | **0.365** | **0%** | European |
| Neoplasm | Vithayathil et al., 2021 | UK Biobank | Head and neck cancer | 1983 | 365578 | 367561 | 312 | SD | 0.90 | 0.72 | 1.12 |  |  | European |
| Neoplasm | De novo analysis, 2021 | FinnGen | Head and neck cancer | 126 | 218666 | 218792 | 305 | SD | 2.89 | 1.13 | 7.37 |  |  | European |
| Neoplasm |  | **Meta-analysis** | **Head and neck cancer** | **2109** | **584244** | **586353** |  | **SD** | **0.96** | **0.77** | **1.19** | **0.688** | **82%** | European |
| Neoplasm | Benn et al., 2016 | CGPS, CCHS | Lung cancer | 896 | 96562 | 97458 | 5 | 10 kg/m^2^ | 1.32 | 0.31 | 5.54 |  |  | European |
| Neoplasm | Zhou et al., 2021 | TRICL, ILCCO | Lung cancer | 29266 | 56450 | 85716 | 776 | SD | 0.95¶ | 0.87 | 1.03 |  |  | European |
| Neoplasm | Vithayathil et al., 2021 | UK Biobank | Lung cancer | 4231 | 363330 | 367561 | 312 | SD | 1.45 | 1.21 | 1.72 |  |  | European |
| Neoplasm | De novo analysis, 2021 | FinnGen | Lung cancer | 1681 | 217111 | 218792 | 305 | SD | 1.69 | 1.30 | 2.20 |  |  | European |
| Neoplasm |  | **Meta-analysis** | **Lung cancer** | **36074** | **733453** | **769527** |  | **SD** | **1.07** | **0.99** | **1.15** | **0.069** | **90%** | European |
| Neoplasm | Dusingize et al., 2020 | Melanoma GWAS | Melanoma skin cancer | 12874 | 23203 | 36077 | 730 | SD | 1.00 | 0.91 | 1.11 |  |  | European |
| Neoplasm | Vithayathil et al., 2021 | UK Biobank | Melanoma skin cancer | 5691 | 361870 | 367561 | 312 | SD | 0.93 | 0.81 | 1.06 |  |  | European |
| Neoplasm |  | **Meta-analysis** | **Melanoma skin cancer** | **18565** | **385073** | **403638** |  | **SD** | **0.98** | **0.90** | **1.06** | **0.530** | **0%** | European |
| Neoplasm | Benn et al., 2016 | CGPS, CCHS | Non-melanoma skin cancer | 7406 | 90052 | 97458 | 5 | 10 kg/m^2^ | 0.71 | 0.26 | 1.93 |  |  | European |
| Neoplasm | De novo analysis, 2021 | FinnGen | Non-melanoma skin cancer | 10382 | 208410 | 218792 | 305 | SD | 0.83 | 0.73 | 0.94 |  |  | European |
| Neoplasm |  | **Meta-analysis** | **Non-melanoma skin cancer** | **17788** | **298462** | **316250** |  | **SD** | **0.86** | **0.77** | **0.95** | **0.005** | **0%** | European |
| Neoplasm | Vithayathil et al., 2021 | UK Biobank | Thyroid cancer | 384 | 367177 | 367561 | 312 | SD | 1.17 | 0.70 | 1.94 |  |  | European |
| Neoplasm | De novo analysis, 2021 | FinnGen | Thyroid cancer | 989 | 217803 | 218792 | 305 | SD | 0.88 | 0.63 | 1.23 |  |  | European |
| Neoplasm |  | **Meta-analysis** | **Thyroid cancer** | **1373** | **584980** | **586353** |  | **SD** | **0.96** | **0.73** | **1.27** | **0.768** | **0%** | European |

**Abbreviations:** ALS, ALS (amyotrophic lateral sclerosis) Variant Server; BEAGSS, Barrett's and Esophageal Adenocarcinoma Genetic Susceptibility Study; BCAC, Breast Cancer Association Consortium; CARDIoGRAMplusC4D, Coronary ARtery DIsease Genome wide Replication and Meta-analysis plus The Coronary Artery Disease; CGPS, Copenhagen General Population Study; CCHS, Copenhagen City Heart Study; CI, confidence interval; COPD, chronic obstructive pulmonary disease; DIAGRAM, DIAbetes Genetics Replication And Meta-analysis; ECAC, Endometrial Cancer Association Consortium; GECCO, Genetics and Epidemiology of Colorectal Cancer Consortium; GERD, gastroesophageal reflux disease; GUGC, Global Urate Genetics Consortium; IGAP, International Genomics of Alzheimer's Project; HERMES, Heart Failure Molecular Epidemiology for Therapeutic Targets; HUNT, Trøndelag Health Study; INVENT, International Network on Venous Thrombosis; IBD, inflammatory bowel disease; IMSGC, International Multiple Sclerosis Genetics Consortium; ILCCO, International Lung Cancer Consortium; ISGC; International Stroke Genetics Consortium; LB, lower bound of the 95% confidence interval.; MDCS, Malmö Diet and Cancer Study; MPP, Malmö Preventive Project; MVP, Million Veteran Program; NA, not available; NAFLD, nonalcoholic fatty liver disease; NHL, non-Hodgkin’s lymphoma OCAC, Ovarian Cancer Association Consortium; OR, odds ratio; PRACTICAL, Prostate Cancer Association Group to Investigate Cancer Associated Alterations in the Genome; RA, rheumatoid arthritis; SNPs, single-nucleotide polymorphisms; TRICL, Transdisciplinary Research Into Cancer of the Lung; UB, upper bound of the 95% confidence interval. *Externally weighted allele score. †1 SD in this population corresponded to 2.76 kg/m^2^. ‡Osteoarthritis diagnosis and osteoarthritis joint replacement (the association was stronger when restricted to osteoarthritis diagnosis only). §This estimate is reversed as results were reported per 1 kg/m^2^ *lower* body mass index in the article. ¶Smoking-adjusted estimate.
